# Uncertainty of Monetary Valued Ecosystem Services – Value Transfer Functions for Global Mapping

**DOI:** 10.1371/journal.pone.0148524

**Published:** 2016-03-03

**Authors:** Stefan Schmidt, Ameur M. Manceur, Ralf Seppelt

**Affiliations:** 1 UFZ, Helmholtz Centre for Environmental Research, Department Computational Landscape Ecology, 04318 Leipzig, Germany; 2 Caprion Proteome, Montréal, QC H2X 3Y7, Canada; 3 iDiv, German Centre for Integrative Biodiversity Research, 04103 Leipzig, Germany; 4 Institute of Geoscience & Geography, Martin-Luther-University Halle-Wittenberg, 06099 Halle (Saale), Germany; Università di Genova, ITALY

## Abstract

Growing demand of resources increases pressure on ecosystem services (ES) and biodiversity. Monetary valuation of ES is frequently seen as a decision-support tool by providing explicit values for unconsidered, non-market goods and services. Here we present global value transfer functions by using a meta-analytic framework for the synthesis of 194 case studies capturing 839 monetary values of ES. For 12 ES the variance of monetary values could be explained with a subset of 93 study- and site-specific variables by utilizing boosted regression trees. This provides the first global quantification of uncertainties and transferability of monetary valuations. Models explain from 18% (water provision) to 44% (food provision) of variance and provide statistically reliable extrapolations for 70% (water provision) to 91% (food provision) of the terrestrial earth surface. Although the application of different valuation methods is a source of uncertainty, we found evidence that assuming homogeneity of ecosystems is a major error in value transfer function models. Food provision is positively correlated with better life domains and variables indicating positive conditions for human well-being. Water provision and recreation service show that weak ownerships affect valuation of other common goods negatively (e.g. non-privately owned forests). Furthermore, we found support for the shifting baseline hypothesis in valuing climate regulation. Ecological conditions and societal vulnerability determine valuation of extreme event prevention. Valuation of habitat services is negatively correlated with indicators characterizing less favorable areas. Our analysis represents a stepping stone to establish a standardized integration of and reporting on uncertainties for reliable and valid benefit transfer as an important component for decision support.

## Introduction

Goods and services obtained from nature–ecosystem services (ES)–are essential for human well-being [1, 2]. Many ES are common goods whose value is often underestimated or ignored in commercial markets [3] and decision making processes [4]. This puts natural capital at risk due to possible mismanagement [1]. Proponents of economic valuation argue that with the quantification of ES in monetary terms conservation strategies and economic objectives could be harmonized, decision-makers better informed and ultimately environmental degradation reduced. Economic valuation of ES is lively debated [5, 6], or even substantially criticized [7, 8]. Arguments for the estimation of ES in monetary terms are that monetary values combine a variety of interdisciplinary measurements in one unit, they are understandable and easily to communicate, and promise transferability across sites [9, 10]. Monetary valuation is seen as a powerful tool for decision making [11], in particular in developing countries [12]. Also it holds the promise of providing an efficient use of limited funds for conservation and restoration [13]. Besides ethical [8] and conceptual concerns [7], there is substantial scepticism that monetary valued ES are globally comparable and reliable, due to the high diversity in human-environment system and the multifarious socio-ecological linkages that influence the perception of societal groups for and finally values attached to ES [6, 14–17].

Despite this critique ES are valued in prominent assessments of natural capital [1, 18, 19], in activities of economic development and poverty reduction [20–22], hazard mitigation programs [23] and business studies [24, 25]. Meanwhile a considerable range of monetary values of ES became apparent across the globe [13]. In primary valuation studies, i.e. first-hand monetary appraisal of ES, effects arising from site- and study-crossing factors are frequently neglected. So are covariates that characterize the context of the study site assumed as being constant and are often not reported in primary valuation studies [26]. Secondary valuation approaches, such as benefit transfer, estimate values for unsampled areas utilizing results from distant studies. Benefit transfer thus aims at putting individual studies in a broader context and is promised to be more time and resource efficient than conducting primary studies [27].

A first major critique refers to benefit transfer in its basic form. Benefit transfer averages monetary values (point estimates) from study sites and transfer them to a similar unsampled area by accounting for land use/land cover types only [13, 28, 29]. More sophisticated benefit transfer approaches, such as meta-analytic value transfer functions control for differences between sites and aim at minimizing errors that come with the transfer process [10]. In any case, assigning a monetary value on nature is not considered to be absolute, rather it is an indication in a particular area, over a given time period, for a specific beneficiary group, depending on valuation context and use. Thus, the crucial question arises: How reliable are value transfer approaches and what are the associated uncertainties?

A second critique originates from the complexity and heterogeneity of human-environmental systems. Due to the variation in site characteristics, e.g. socio-economic or biophysical feature [30, 31], and study characteristics, e.g. valuation method [32, 33], the error resulting from generalization and transfer is the core critique. In order to apply benefit transfer models for decision making it is required to identify potential errors [26, 34, 35], establish an accepted framework for assessing the magnitude of errors and incorporate the uncertainties to the formal valuation process, as well as communicate monetary values directly in association with uncertainties to decision makers. Therefore, the second key question of our study is: What promises in transferability of monetary valuation of ES can be hold given most up-to date data?

With this publication we i) provide a conceptual base supporting the establishment of a standardized integration and reporting on uncertainties of benefit transfer. Building on this we ii) assess the transferability of monetary values of ES and identify major sources of uncertainty by using meta-analytical value transfer functions.

We generated a spatially explicit database of 194 globally distributed cases studies covering 839 monetary values of ES from peer reviewed data collections [13, 36]. We built robust meta-analytic value transfer functions and tested the importance (statistical influence) of 93 site- and study-specific covariates in explaining the variability of monetary valued ES. This allows us to identify key sources of uncertainty of the value transfer functions at finer spatial scale (30 arc min). In doing so, we conducted the first comprehensive uncertainty analysis for a set of twelve monetary valued ES on a global scale. Findings in our analysis show i) the first global valuation map in monetary terms based on meta-analytic value transfer functions, and ii) crucial parameter and uncertainty that needs to be considered to lower transfer errors.

## Methods

### Synthesizing databases on monetary values

**Fig 1:**
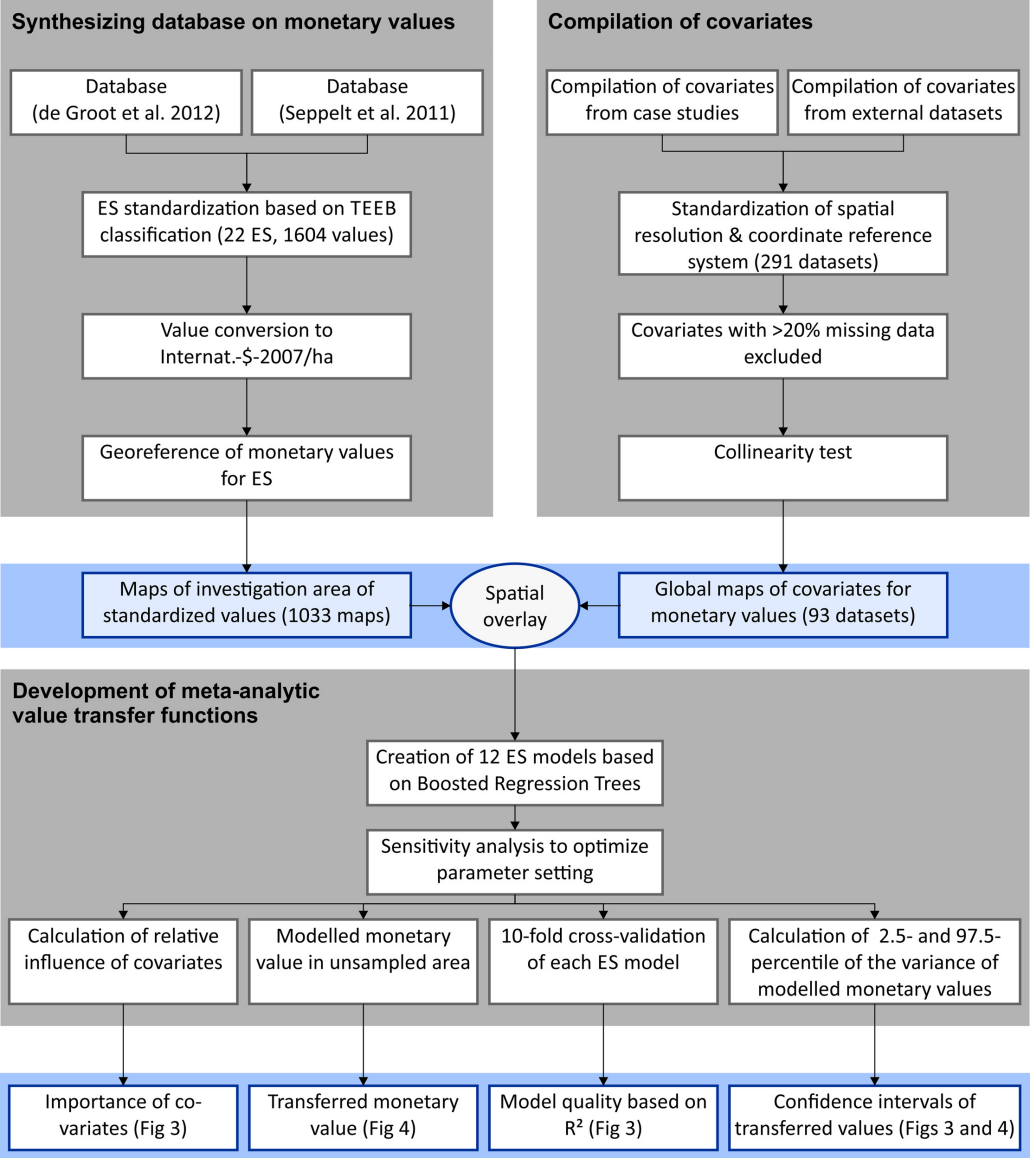
Workflow from data compilation to uncertainty estimation. The diagram shows different steps of data preparation and analysis (grey boxes): i) synthesis of monetary values (response variable), ii) compilation of covariates that are supposed to affect the variance of monetary values; and iii) development of value transfer functions. The bluish boxes show (interim-) results of the different steps and refer to figures that visualize these outputs.

We combined two, peer reviewed databases with monetary valued ES [13, 36], see Fig 1, S1 Table. ES were harmonized to a common, comparable set of ES types using a standardized classification system [37] to avoid semantical differences between varying ES terminologies. In this standardized classification 22 ES types are grouped in four classes: provisioning, regulating, cultural and supporting ES. Monetary values were translated into 2007 “International Dollar” per hectare and year by using the World Bank deflator and purchasing power parity conversion factors [13]. Furthermore, we extracted from the case studies detailed information on the investigation areas for each ES type (S2 Table) and used ArcGIS 10.2.2 in order to geo-reference the study site spatially explicit. In total 1033 maps of standardized monetary values were generated.

### Compilation of covariates

Based on a literature review of variables that are supposed to affect the monetary valuation we identified relevant global geo-datasets. These data were used as explanatory variables for the statistical analysis and will be hereinafter referred to as covariates (Fig 1). Data captures six groups: economy, policy/governance, other societal data, ecology, valuation methods, and scale. Some of these covariates were derived from the original studies. S2 Table provides an overview. All covariates were standardized to the same coordinate reference system (WGS 1984) and same resolution of 30 arc min, see Fig 1. If spatial coverage shows more than 20% missing values, covariates were excluded from the analysis. Also covariates were tested for collinearity and highly correlated excluded. For continuous covariates we used Pearson correlation coefficient (***r*** >0.75 or ***r***< -0.75*)* and Spearman’s rho (***ρ***>0.75 or ***ρ***< -0.75), for two categorical covariates chi-square test and for testing categorical and continuous one-way ANOVA. From 291 covariates 93 remaining were combined with the maps of standardized monetary values by overlay operation in ArcGIS. Combined maps were used as input for the value transfer functions (Fig 1). This data collection is one of the most comprehensive databases available.

### Meta-analytic value transfer functions

We computed meta-analytic value transfer functions for each ES type using additive regression models based on boosted regression trees (BRT). A BRT computes the relative influences (importance) of covariates for a BRT model, i.e. they identify major determinants of the variance in monetary values of ES. BRT also provide elasticity curves (partial dependence plots) that account for non-constant marginal value changes over distinct socio-ecological conditions and thus quantify the change of monetary values in response to an alteration in one covariate (i.e. ceteris paribus) [38]; see S2 Fig. Resulting BRT models were used to transfer and map values in unsampled areas. BRT are specifically suited to quantify comprehensible covariates in situations where many variables are expected to explain the process at hand, which interact in complex, non-linear ways [39]. They allow for including different types of covariates (numerical, binary, categorical), can accommodate missing data in covariates by using surrogates [40] and show high robustness to the effects of extreme outliers. BRT models were computed utilizing the Generalized Boosted Regression Models library [41, 42] with the programming language R [43].

We tested different parameter of the BRT algorithm such as learning rate, tree complexity, minimal number of observations in terminal nodes and number of trees (see S1 Text), and choose a robust model with high explanatory power. The final parameters selected for the BRT models are documented in S3 Fig.

Statistical significant value transfer functions could be computed for 12 ES types based on 839 monetary values (out of 1033 monetary values). Most important covariates were quantified and monetary values in unsampled areas extrapolated (Fig 1). If there were less than 11 case studies or less than 26 monetary values data were not sufficient to generate reliable value transfer functions (for 10 out of 22 ES). We globally mapped monetary value for 12 ES types on a 30 arc min grid by applying the derived value transfer functions based on global covariates for the entire terrestrial earth surface (except the ant- and artic areas). For the spatial value transfer we used the R library Raster. Moreover, we computed the coefficient of determination R-squared for each value transfer function based on ten-fold cross-validation to estimate the explanatory power.

In an additional step confidence intervals were estimated to examine generalization failure of value transfer functions from training data (Fig 1). We rerun the BRT models under different parameter settings (see S1 Text), calculated the 2.5- and 97.5-percentile values of the variance of transferred monetary values and mapped the range of percentiles for each grid cell. Three classes of uncertainty (low, middle, high) were used for mapping based on equal-interval classification for each ES separately. Finally, 12 bivariate maps were created by overlaying the classes of uncertainty with maps of extrapolated monetary values mentioned above. These maps were used to estimate the percentage area of terrestrial earth surface covered by transferred values of low, middle and high uncertainty (Fig 1).

For the discussion of the results we conceptualized three major sources of uncertainty of value transfer functions: (i) Sample error, such as measurement error in input studies for value transfer functions or publication selection bias; (ii) errors originating from statistical estimation of BRT models (model performance and suitability of chosen approach for benefit transfer); and (iii) transfer error from generalization that encompasses distortions due to value transfer without fully accounting for site and study characteristics. Only covariates with >1% (relative contribution for value transfer functions) were analyzed for the six groups of covariates. Additionally, in a fourth point we provide information of the spatial application of the value transfer functions.

## Results

### Overarching findings

#### Available input data

**Fig 2:**
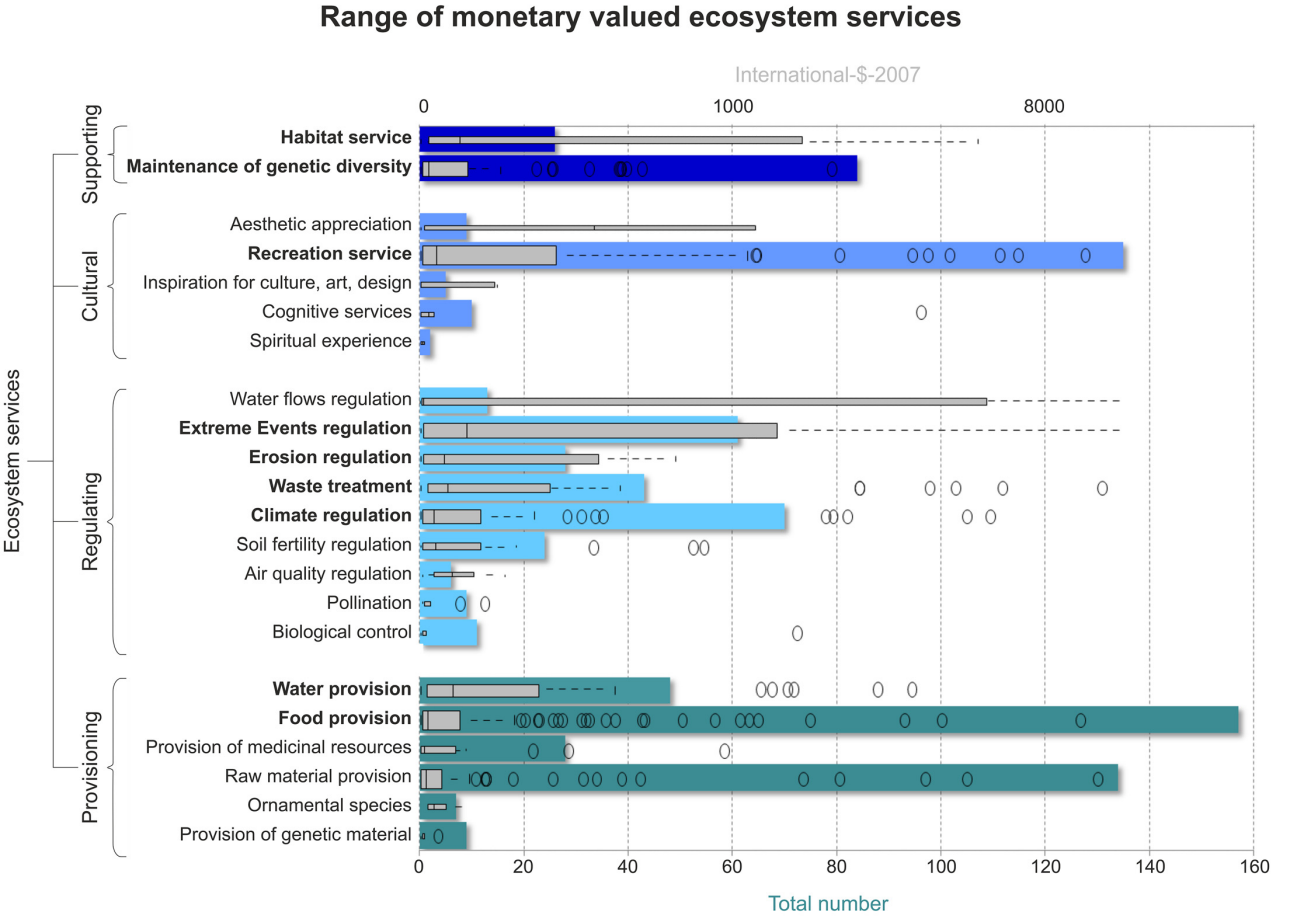
Range of monetary valued ES. Represents the database of unit-adjusted monetary values of standardized ES types [37] from peer reviewed data collections [13, 36]. The coloured bar charts reflect the total number of monetary values per ES type; the grey boxplots represent the variability of economic values. ES in bold font indicate the selection of 12 ES types (839 values) that were used for the value transfer functions.

All studies considered in the synthesized database (Fig 2) come from scientific peer reviewed publications (48%) or grey literature selected by experts with background in ecological economics (52%) [13, 36]. The majority of the studies were conducted in lower latitudes, i.e. areas with an annual mean temperature >15°C (63–97% of case studies per ES), areas with in average more than 100 inhabitants per square kilometer and high accessibility to international markets (89–97%), as well as areas with high threats of degradation (59–83%). In only 7% of studies investigation were carried out in countries with a per capita income smaller than 1045 International-$-2005 (purchasing power parity adjusted).

#### Model uncertainty

**Fig 3:**
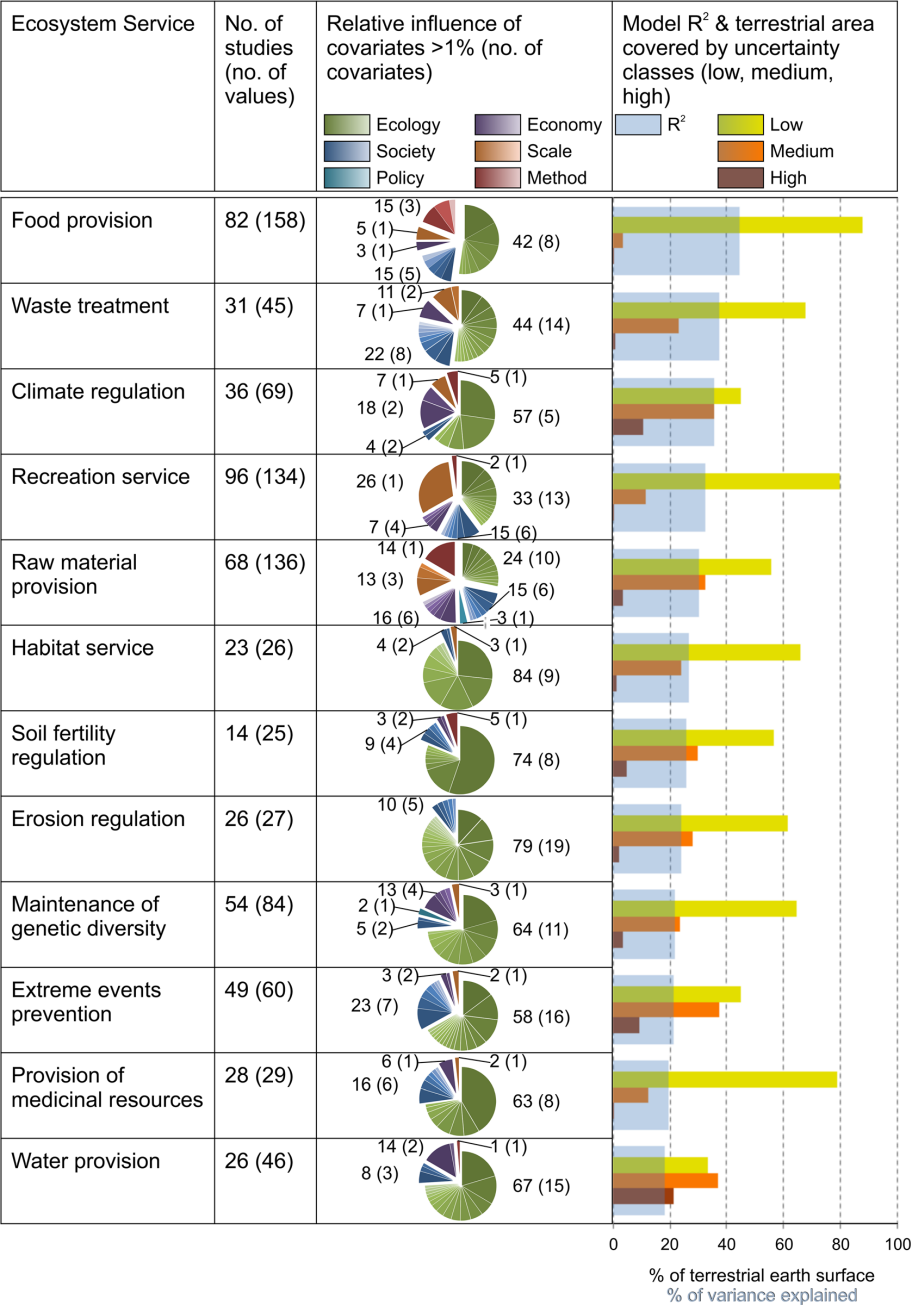
Overview of input data and characteristics of value transfer functions for 12 ES. The table shows for each ES the number of case studies and monetary values (2^nd^ column). In the 3^rd^ column pie charts reflect the relative influence (importance) of groups of covariates expressed in percentage values and number of covariates (number in brackets) in these groups. The importance of covariates is illustrated by the size of the pie slide and quantified in S2 Fig. The bluish bar charts in column 4 represent the model quality based on percentage of variance explained by the model (R-squared). Additionally, column 4 shows the percentage area of terrestrial earth surface covered accordingly to uncertainty classes (low, medium, high).

The final 12 cross-validated value transfer functions explain from 18% (water provision) to 44% (food provision) of the variance of monetary values (Fig 3). Furthermore, confidence intervals for the value transfer functions were calculated which display low to medium uncertainties for 70% (water provision) to 91% (food provision) of the terrestrial earth surface (Fig 3).

#### Importance of covariates

Results from the quantification of the importance of covariates for the value transfer functions indicate which site and study characteristics need to be considered in order to minimize transfer error from generalization. In Fig 3 most influential variables (>1% relative contribution for value transfer function) are shown for six groups of covariates. Ecosystem-based covariates (green) are the most important (up to 90%). Contrary to the frequent critique on the monetary valuation of ES, covariates from the economy (purple, 1%-19%) or from other societal and policy settings (dark- and light blue, 6%-27%) show lower influence. Also covariates describing the analytic dimensions of scale (orange, 1%-26%) and valuation methods (red, 0–15%) are relevant, but contributing little to the explanation of the variance. Subdividing the groups of covariates show that variables indicating environmental degradation are most influential, followed by the variables of spatial extent of investigation area from case studies and market accessibility.

#### Application of value transfer function

**Fig 4:**
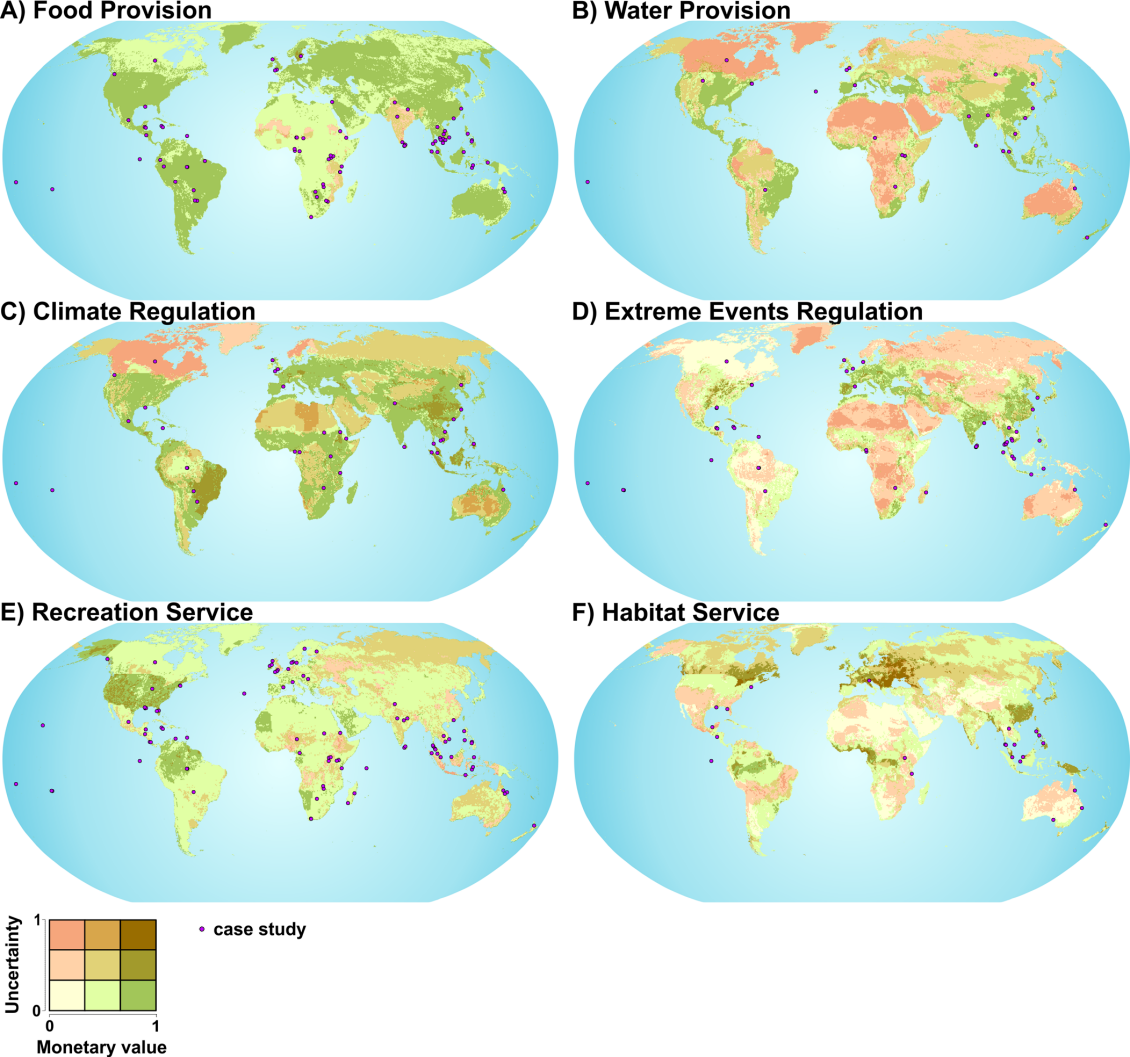
Global spatial distribution of monetary estimates and uncertainties. The bivariate maps show the extrapolated relative monetary values (yellow to green) and uncertainties (yellow to red) of the meta-analytic value transfer functions for the ES: A) food provision, B) water provision, C) climate regulation, D) extreme events regulation, E) recreation service and F) habitat service. Monetary values and uncertainties are grouped into three classes (low, medium, high) accordingly to the spatial extrapolations of the optimized value transfer functions respectively the confidence intervals of transferred monetary values (see method section). The classes were defined by equal interval distances for each ES separately. Accordingly, classes between ES contain different ranges of values. However, a standardized color code (0–1) was used for simplicity of visualization.

The application of the value transfer function for spatial extrapolation of values in unsampled areas results in a map shown in Fig 4. Summarizing findings across all ES show that highest uncertainties are computed in areas where no case studies are available or covariates are missing. These are sparsely populated areas such as big deserts (Sahara, Kalahari, Desert of Australia, Arabian Desert), taiga and tundra (parts of Siberia, eastern Canada), ice and snow area (Greenland) as well as highlands (North America).

In the following section we discussed the results in detail for 6 out of 12 ES. Food and water provision, climate and extreme events regulation as well as recreation and habitat service were selected, because they represent the highest variance of monetary values from each ES group (provisioning, regulating, cultural and supporting), see Fig 2.

### Ecosystem service specific results

#### Food provision

Available input data: With 158 monetary values the largest data set is available for food provision. The majority of case studies examine fish provision (51%) in lower latitudes (93%) and in coastal ecosystems or inland wetlands (69%). Lower latitudes (annual mean temperature >15°C) are most likely food-insecure regions with a high vulnerability to climate change [44]. Coastal ecosystems and inland wetlands are among the most human-impacted habitats globally [45, 46].

Model uncertainty: The value transfer function shows the lowest uncertainty and explains 44% of the variance in the data. Estimations of the confidence intervals show that for 91% of the terrestrial earth surface monetary values can be computed with low and medium uncertainty (1 to 80 Int.-$-2007 per ha), due to a high number of data points (Fig 3).

Importance of covariates: Most important covariates are climate indicators (22%), followed by geographic and nature endowment (15%) and valuation methods (15%). Further influential are social covariates such as better life domains of human well-being (12%) and religion (3%) as well as the economic covariate agricultural subsidies (3%).

Ecology: Covariates indicating climate and those on geographic and nature endowment show low values in areas with prevailing unfavourable growing conditions (14%, annual mean temperature >29°C, 7% annual mean moisture index <0.78) and low human-induced alteration of ecosystems (2%, Human appropriation of net primary production (HANPP) <11%) or high amounts of alternative food products (3%, extent of agricultural areas >30km^2^ per grid cell). Variance explained by distance to sea (9%) is a logical consequence from the distribution of studies and the focus on fish as food resource. In more landlocked areas the importance of fish in the food supply reduces and so does the monetary valuation of fish. Moreover, the type of biome explains 6% of the variance, but does only partly coincide with the land use classification used on the case studies itself. These show that coastal wetlands, coastal systems and cultivated areas (aquaculture) are most valued, and confirm previous findings from [13].

Valuation method: Direct market pricing is most often used for monetary valuation of food (70%), followed by benefit transfer (17%) and group valuation (5%). With group valuation significant lower values are derived. Most studies (75%) based on group valuation were conducted for fish in India, a country where fish consumption represents only 2% of protein intake [47], which might explain the lower valuation. Also the value type calculated in the case studies influences the variance of monetary valuation. Annual values (91% of valuation) are systematically lower than one time payments (3%) and net present values (3%). The latter two value types consider more complex ecological and economic features, which may explain the higher values. Apart from fish (51%) also plants/vegetable food (15%) and non-timber forest products (15%), unspecific food (14%) and meat (5%) were valued. The ES subtypes explain 6% of the variance. Fish is highest valued and, on the contrary, meat lowest. Case studies focusing on the provision of meat were all conducted in developing countries, where starchy staples (e.g. maize, manioc, millet) are major part of the diets.

Society: The valuation of food increases with a lower unemployment rates (<10% of labour force) and a high number of people in working age (15–59 years), good education system (loss of schooling years due to inequality <30%) as well as with improving sustainable well-being measured by years of life satisfaction achieved per unit of resource used (Happy Planet Index). These covariates directly relate to the better life domains of the Organisation for Economic Co-operation and Development (OECD), which are essential metrics for human well-being [48]. This shows that the more positive the conditions for human well-being the higher people value food. This might be contra-intuitive, as people who desperately rely on external support for food and are undernourished would value food much higher. These regions, however, are not captured by the available data sets. We also found that the religious confession influence monetary values. Particularly in major Hindu sites significant lower values for food can be identified. Although, the dietary standards of Hindus vary in time and place, most of them do not eat fish. Except Hinduism the majority of the world religions are predominantly non-vegetarian [49].

Economy: With increasing subsidies in agricultural sector the value for food provision decreases. Subsidies distort markets by promoting the production of agricultural commodities beyond market demand, thus, they encourage farmers and fishermen to rely on them instead of consumer wants [50].

Application of value transfer function: Spatial extrapolation of values shows for food the highest uncertainties and lowest values are in India and parts of Africa (Fig 4A). On contrary, most certain and highest values are in China, South-East Asia, USA, Brazil, Mexico, EU-member states and parts of the Russian Federation. It is striking that these areas match regions where a high consumption of fish and fish products as well as high capture rates occur [47].

#### Water provision

Available input data: For water provision one of the smallest datasets was available (26 case studies). Most of the case studies were conducted in climate sensitive lower latitudes (63%). Climate change is affecting the hydrologic cycle and directly impacts the water resource base, usage, and management, in particular in lower latitudes [51].

Model uncertainty: The value transfer function for water provision shows the highest uncertainties and explains 18% of the variance (Fig 3). Confidence intervals of transferred values illustrate that only 70% of the terrestrial earth surface is covered by low and medium uncertainty classes (1 to 26 Int.-$-2007 per ha).

Importance of covariates: Most influential are ecosystem-based covariates that indicate biodiversity and water availability (67%), followed by type of biome (3%). Further important are covariates of social and economic indicators (22%).

Ecology and society: Areas of high biodiversity threat and conservation value are positively correlated with monetary values and explain 21% of the variance. Biodiversity and water supply are strongly interrelated [52, 53]. Drivers leading to biodiversity loss, such as pollution or river fragmentation, are the same that causing water security problems [54]. Sites with high biodiversity show high human populations and substantially higher human population growth rates than that for the entire world [55, 56]. Therefore, increasing population drives the value for water (8%). These patterns can also be found in the spatial results of our value transfer function (Fig 4B), see below. Furthermore, in conservation areas the sensitivity of beneficiaries for the protection of common goods is more pronounced. We can find further drivers which put water provision under pressure and are important for the value transfer function. First, a higher risk of erosion leads to higher values (12%). Second, increasing anthropogenic altered habitats due to land use change and harvest of primary production (8%, HANPP >15%, pasture area >5km^2^ per grid cell), in particular, agricultural frontier areas of cropland foster high values for water provisioning (4%, crop area between 3 and 20km^2^ per grid cell). Third, deforestation explains 3% of the variance and is positively correlated with water values. Water availability, moreover, depends on the spatial (3%) and temporal allocation of water resources (2%). The more unequal rivers and lakes are distributed in an area the higher is the value for water provision. Seasonal variability or long-term climatic changes cause extended periods of droughts or water abundance. The type of biome explains 3% of the variance. However, the applied land use classification system differs from the actual biome type of the case studies. Taking case study based classification into account coastal wetlands and freshwater (rivers/lakes) show highest values, confirming [13, 28, 29].

Economy: The portion of privately owned forests is positively correlated with water provision value (5%). This confirms that weak ownerships might affect valuation of other common goods negatively [57]. Similarly, there is a strong relationship of higher tax revenues which leads to lower values of water services (12%) pointing out to the fact, that with higher economic activities, more technical solution which can provide access to water are more likely to implement, because technical substitution is affordable [54]. We further found negative correlation between values and renewable energy production (2%).

Application of value transfer function: Spatial extrapolation of values shows the most certain and highest values in areas of high population growth and increasing pressures on water security, for instance in China, India, Java, eastern USA, south Mexico and Western Central America, south coast of Western Africa, and Mediterranean Europe (Fig 4B). Transferred values with the highest uncertainties coincide with the lowest values and appear in areas where almost no case studies are available. Most of these areas represent the big deserts of the earth (Sahara, Kalahari, Namib, Australian, Arabian, Thar, Dasht-e Lut, Karakum, North American), eastern central Africa, Siberia, Greenland and Canada.

#### Climate regulation

Available input data: From a total of 36 case studies (69 monetary values), carbon sequestration was most frequently examined (68%), followed by other greenhouse gases (<2%) or remain unspecified (24%). The majority of studies were carried out in tropical and temperate forests (25%) as well as inland and coastal wetlands (45%). These biomes are seen as regions highly suitable for carbon sequestration [58, 59]. Moreover, most studies are located in climate sensitive, lower latitudes (72%), similarly to the ES mentioned before.

Model uncertainty: With 38% of variance explained in monetary values the transfer model for climate regulation represents the third best prediction performance (Fig 3). For most of the terrestrial earth surface (81%) confidence intervals could be calculated that show low to medium uncertainty (1 to 21 Int.-$-2007 per ha).

Importance of covariates: The most important covariates are ecosystem-based variables of nature threats (49%). Also relevant but explaining less of the variance are covariates indicating input measures of climate sensitive economic sectors (20%) and other economic variables (6%), followed by scale (7%), methods (6%) and social variables (4%),

Ecology and economy: Covariates of nature threats show that high values are associated with areas of high risk that unique biodiversity will soon be lost (30%) and high risk of erosion (19%). Covariates indicating input measures of climate sensitive economic sectors (e.g. energy production and water business) are positively correlated with monetary values for climate regulation. Most important covariates are: proportion of electricity production from hydroelectric sources (12%), annual mean of solar radiation (6%) and the water storage capacity of dams per country (2%). Values are high, for instance, in areas of lower latitudes where up to 99% of electricity is produced by hydroelectric power plants and where big artificial constructed water reservoirs (dams with capacity of < 3000 km^3^) exist. Furthermore, the economic covariate inequality of income (6%) shows that the more unequal income is distributed the higher the value for climate regulation.

Scale and valuation method: Spatial extents of investigation areas as well as valuation methods applied in the case studies explain respectively 7% and 6% of the variance. With a greater spatial extent monetary values decrease. These diminishing returns may occur because of declining marginal utility for beneficiaries. The majority of studies based on benefit transfer (60% of studies), followed by direct market prices (16%) and avoided costs (11%). For direct market prices the highest values and for benefit transfer and replacement costs the lowest ones can be observed.

Society: Social covariates of relevance are population density per country (2%) or the age of population (2%). Population density is negative correlated with monetary values. Surprisingly, case studies in countries with on average older population report higher values of climate regulation. One might hypothesize older people may have made in their lifetime perceiving changes in climatic conditions and associated consequences, thus, valuing climate regulation service higher than younger persons. This might be an indication for the “shifting-baseline” hypothesis, i.e. shifts of the reference points of human perception for estimating changes [60].

Application of value transfer function: Applying the value transfer function shows that the most certain and highest values are computed for areas under threat of habitat alteration due to climate change or other land degradation processes. Examples are areas like the Sahel Zone, tropical islands and mountains, Mediterranean ecosystems, Eastern USA and parts of Europe (Fig 4C). On contrary, abandoned areas with the highest suitability of soil for carbon sequestration, such as Canadian and Siberian Tundra and boreal forests cover most uncertain and lowest values. This is due to a lack of case studies in such regions.

#### Prevention of extreme events

Available input data: The majority of case studies (82%) were conducted in areas of high vulnerability to extreme events and sites which are increasingly exposed to extreme events due to climate change [61–63]. In 42% flood prevention was considered, followed by unspecific extreme event prevention (30%), storm prevention (20%) and fire prevention (3%).

Model uncertainty: The value transfer function for prevention of extreme events represents medium prediction performance and explains 21% of the variance in monetary values (Fig 3). Transferred values of low and medium uncertainties (1 to 26 Int.-$-2007 per ha) are calculated for 82% of the terrestrial earth surface.

Importance of covariates: The impact of extreme events depends on both ecological conditions and societal vulnerability. We found that with 64% the highest explanatory power is represented by covariates showing the inherent conditions of ecosystems, followed by socio-economic characteristics (33%) and covariates dealing with the spatial and temporal scale (3%).

Anthropogenic pressure on the ecosystem: Extreme event prevention is valued high in areas with advanced anthropogenic alteration of ecosystems characterized by highly changed biomass (HANPP 13%), high agricultural induced soil erosion rates (10%), degraded freshwater resources and riverine biodiversity (10%), dense infrastructure (5% market access), dense settlements with major markets (4% Anthromes) and high population density (3%). Hence, the risk awareness to weather extremes and natural hazards increases with number of people potentially effected and higher level of land-use and degradation. The more risk aware a society is, the more weight it places on strategies that preserve or build ecosystem resilience, and the higher the value it would allocate to ecosystem configurations that are more robust [64]. This is further emphasized by our findings that monetary values are high in nations seeking for sustainable political governance, i.e. protect ecosystems and manage productive natural resources efficient for both economic growth and for supporting human well-being, while reducing environmental harm (2% adjusted net savings, 1% Environmental Performance Index, 1% Happy Planet Index).

Species richness and unemployment: We also found that areas with high species richness (4%) and high level of conservation priority (3%) show high valuation for extreme event prevention. These areas are characterized by high population, the highest population growth rates globally [55, 56] and economically poorest societies [65]. In nations, moreover, with a high unemployment rate (11%) extreme event prevention is valued high. Societal vulnerability to extreme events arises from the inability of people to withstand adverse impact from extreme events. Poor people have little adaptive capacities due to limited technological mitigation options, such as building codes and disaster preparedness [66]. This increases their exposure to severe adverse consequences of extreme events; particularly in areas with anthropogenic altered extreme event buffers. A variety of species may provide valuable insurances against severe disruptions [67].

Application of the benefit transfer function: Using the value transfer model for spatial estimations of extreme event prevention show that the most certain and highest valuations are situated in densely populated areas with advanced anthropogenic altered environments (Fig 4D). The largest uncertainties are associated with low values and are shown in low populated areas where case studies are missing. Similarly to the findings from water provision, these areas are the big deserts of the earth (Sahara, Kalahari, Namib, Australian, Arabian, Thar, Dasht-e Lut, Karakum, Northamerican), eastern central Africa, Siberia and Greenland.

#### Recreation

Available input data: The database for the ES recreation services captures with 96 the most case studies. The majority of studies were conducted in major non-religious and Christian sites (73%) as well as lower latitudes (77%). Studies in developing countries (Human Development Index < 0.6, gross domestic product (GDP) < 4024 US-$) are underrepresented (26%). Subcategories of recreation services investigated are tourism (41%), hunting (<1%), ecotourism (<1%) and unspecific recreation (47%).

Model uncertainty: The value transfer function for recreation services is with 33% variance explained the fourth most certain model (Fig 3). Confidence intervals show that low to medium uncertainty (1 to 26 Int.-$-2007 per ha) could be extrapolated for 91% of the terrestrial earth surface.

Importance of covariates: With 32% ecosystem-based covariates are the most influential group. In comparison with other value transfer functions, however, this is the lowest value (Fig 3). Further important covariates are those representing the spatial extent of investigation (25%), socio-economic variables (22%) and methods used (2%). The spatial extent of investigation is the most important variable in the transfer model and negatively correlated with monetary values.

Ecology: More homogeneous environments receive lower values for recreation. The value transfer function shows that areas are low valued with intensive agricultural use (6%), a high number of people living on degraded land (2%) and low number of terrestrial protection areas (4%) and biodiversity (2%) as well as low ethnic diversity (1%). Surprisingly there is a negative effect of marine protection areas (4%) and length of coastlines (1%). This is due to the low number of case studies carried out in coastal areas (17%) and marine protection areas (16%). Furthermore, covariates on land use/land cover types explain 9% of the variance and show that wetlands and freshwater areas (river/lakes) are highest valued. The most important climatic covariates are solar radiation (3%) and soil moisture (2%). The latter one is strongly correlated with precipitation. Highest monetary values are shown for areas with 100 to 190 W/m^2^ and a soil moisture index of 1 to 1.6. This corresponds to areas in the (sub-) tropics, see Fig 4E. Climate effect human’s psychological perspective as the beneficiary enjoys, for instance, sunshine/cloudiness, hours of daylight, UV radiation (health, suntan) [68]. Covariates, however, confirm too less or too much radiation or humidity can also reduce attractiveness, e.g. short day length (depression, vitamin deficiency) or very high UV radiation (sunburn, allergies).

Economy and society: Economic covariates show that recreation is highly valued in wealthy countries. These countries are characterized by high market access (3%), low GDP growth (3%), low consumer prices (2%), positive trade balance and a high level of export to import ratio (3%). In wealthy countries the access to a greater share of ES and substitutes is provided, thus, the basis for recreation given. However, in areas with a high unemployment rate (6%) recreation values are high, too. We hypothesize these are sites where tourism is already established and an important economic factor. With increasing portion of privately owned forests the value for recreation is higher (1%). This shows that with the granting of property rights the valuation of common goods might be positively affected [57] and emphasize findings we made for food provision.

Application of value transfer function: Applying the value transfer function shows that the most certain and highest values are in the USA, most of the Mediterranean area, Caribbean islands, and Colombia (Fig 4E). These areas coincide with most attractive tourist places mentioned by the World Tourism Organization [69]. Highest uncertainties and simultaneously lowest values are in the Russian Federation, African regions along the Sahel Zone, Indonesia and parts of Australia.

#### Habitat service

Available input data: The database for habitat service contains one of the smallest numbers of case studies (23 case studies, Fig 3) and highest variances in monetary values (Fig 2). In the majority of case studies (96%) the provision of habitats for young species (nursery) was conducted. Furthermore, most studies (92%) took place in climate sensitive lower latitudes. Habitat services are highly responsive to climate change and substantial alterations expected [70, 71].

Model uncertainty: The resulting value transfer function of the small and highly varying data set shows moderate uncertainty and explains 27% of the variance in the data. For 90% of the terrestrial earth surface values could be extrapolated with medium to low uncertainty (1 to 14 Int.-$-2007 per ha).

Importance of covariates: The group of ecosystem-based covariates contributes with 90% to the value transfer function (Fig 3). These are climate covariates (42%), followed by soil (24%) and water variables (24%), as well as biota (7%). Furthermore, social covariates (4%) and the spatial extent of the investigation area are relevant (3%). Social variables such as ethnicity are also relevant, but influence the value transfer function with <1% only slightly. We assume this is caused by the small number of monetary values.

Ecology and society: Habitat services are valued less in marginal areas, i.e. harsh growing conditions due to low soil quality (24%), arid climate zones with high drought potential (16%) and high UV exposure (26%). Marginal areas are mostly sparsely populated (1%) and characterized by low market access (3%). Also those areas are the home of poor people [72, 73] with little awareness of biodiversity [74] and subsistence economy prevails [72]. Consequently, land management is prioritized in order to meet basic needs, such as food provision or use of raw material. This is supported by the findings that in countries with high proportions of privately forests values for wildlife habitats are low (12%). On contrary, humid areas with increasing soil carbon content representing enhanced habitat quality and high species richness (4%) are positively correlated with monetary values of habitat services. This shows the direct link between habitat function for wildlife and food provisioning. Furthermore it emphasizes the importance to conserve habitat areas as a prerequisite for food and other ES. European studies even show that habitats under conservation (for example in protected areas) provide more regulating and cultural ES than other habitats [75, 76].

Application of value transfer functions: Spatial extrapolation shows the most certain values in low valued marginal areas like the highlands and drylands of Asia and sub-Saharan Africa, transition zones of the grassland to deserts of Australia as well as southern Latin Americas Andes Mountains, and the highlands and drylands of North America (Fig 4F).

## Discussion

**Fig 5:**
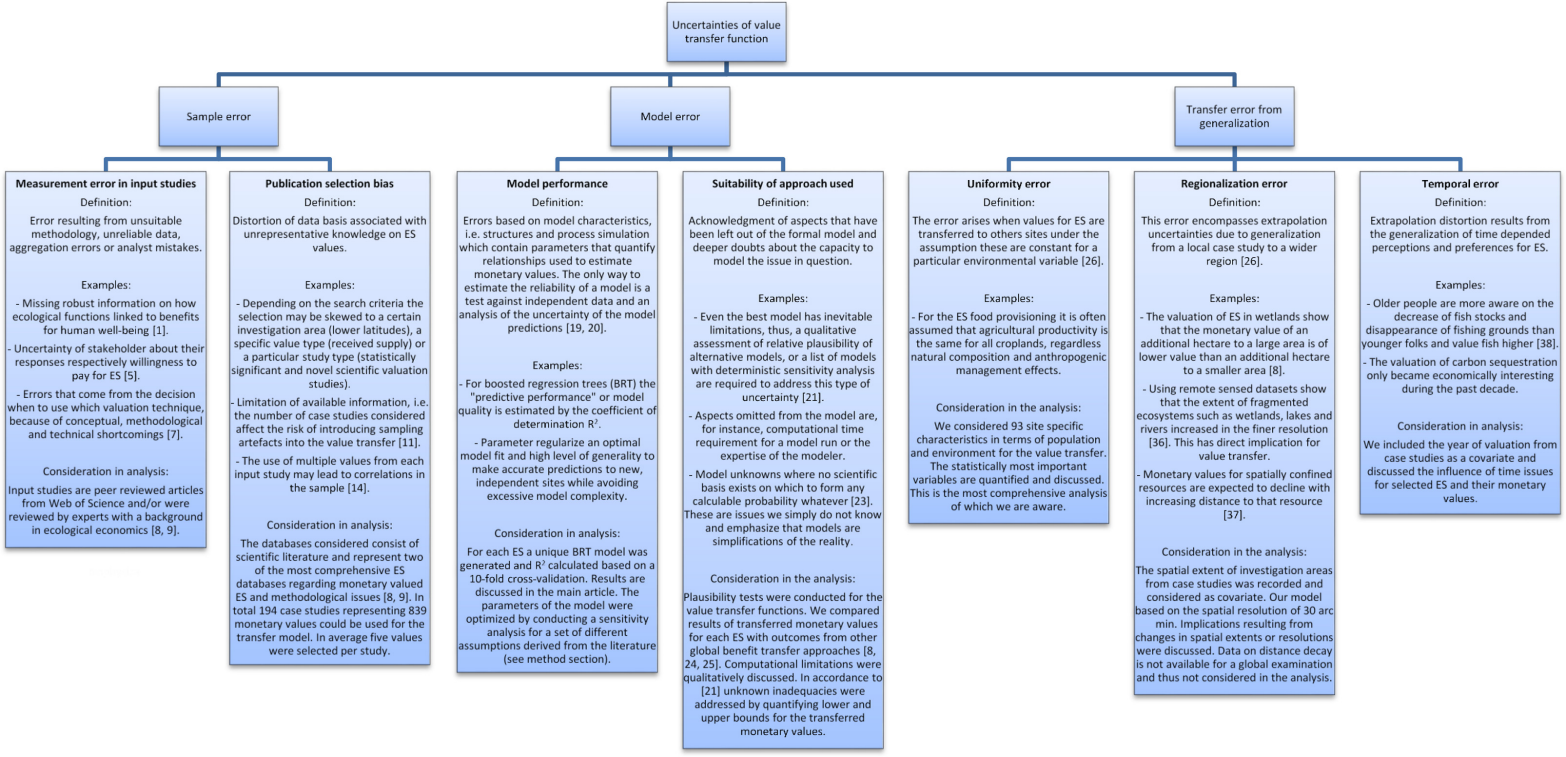
Overview of uncertainties in modelling global value transfer functions. The boxes denote uncertainties, which we either considered directly in the model design or discussed qualitatively.

Although “money” is seen as a well-known and easily understandable indicator, monetary valuation of ES is ambiguous and shows only a fraction of the multiple characteristics that give utility to the beneficiary [77]. Applying benefit transfer and mapping a variety of ecosystems, social preferences and economics constraint to a single monetary value is an aggregation in which information is lost. To compare and transfer values in a reliable and valid manner we determined three major sources of uncertainty: a) the availability and quality of input data; b) the performance and suitability of the estimated transfer model and c) the spatial application of the transfer model in order to estimate global ES value maps; see Fig 5.

The data availability and the quality of input studies determine the sampling error. We only included valuation studies that were reviewed by experts as a minimal assurance of study quality. Although the peer review approach is often criticized it is also appreciated as an efficient method that increased the scientific progress over the past decades [78]. The majority of case studies were conducted in lower latitudes (63%-97% of all case studies). Most of the studies, moreover, were located in areas characterized by a high population density and market accessibility above global average (89%-97%) as well as high threats of degradation (59%-83%). In situations of declining natural capital and continuing high demand, beneficiaries are sensitive to changes in natural capital [38], which might lead to overestimation. We thus assume that the global maps of monetary valued ES, illustrated by Fig 4, represent an upper estimate of ES values, particularly for transferred values in more pristine areas. Furthermore, only 7% of studies were conducted in countries with a per capita income smaller than 1045 International-$-2005. Thus, there is a major research gap in the poorest countries [79] where people use the environment in a more direct way than richer ones and dependence on as well as preferences for ES are likely to be different compared to developed countries [4]. Results of our models confirm that transferred values in poorest areas are associated with high uncertainties (Fig 4).

Another concern with respect to the input data refers to the aggregation of the monetary values. In order to create a comparable data base for spatially explicit value transfer modelling, monetary values need to be disentangled. Only values measured under marginal changes in the socio-ecological system were considered [38]. We utilized units expressed in “estimate per unit area value”, in contrast to “estimate per beneficiary” which are often used for cultural services [80]. The first unit represents supply rather than demand aspects. Specifically, the potential supply rather than the received service is reflected for a given time period. Thus, the values cannot be utilized to identify societal urgent needs [81]. Accordingly, highest valued regions in Fig 4 (and S1 Fig) do not represent areas with potentially highest supply of ES nor the societal most important ES. The maps in Fig 4 may not be used to prioritize the most worthy ecosystems for human welfare, as suggested in [28, 29].

Utilizing BRT for spatially explicit, meta-analytic value transfer functions have inherent causes of error, too. Although this method is acclaimed to be one of the most sophisticated approaches for benefit transfer [28, 82] the evaluation with R-squared (cross-validated) revealed that none of the value transfer functions explain more than 44% of the variance in the data. Reasons for the comparably low explanatory power are the small number of case studies (Fig 3) as well as computational limitations, which originate from the requirements for calculations that limited our spatial analysis. A finer resolution than 30 arc min or discrete predictors with a high number of categories could not be employed in the statistical analysis. Higher spatial resolution data might provide better results, but the limiting factor of model performance is the small number of case study data. Further a model fitting the data with a high R-square (postdiction context) may not generalize well (prediction context). Thus using new modelling techniques (BRT, random forests, etc.) should complement a search for more data.

The spatial application of the value transfer functions in unsampled areas generates transfer errors from generalization, which are determined by uniformity, spatial extent and resolution (regionalization error) as well as temporal aspects [4, 83]. We found that ecosystem related uniformity errors are the key source of potential error in benefit transfer. Uniformity error occur if values for ES are transferred under the assumption that important covariates are constant [83]. For food provisioning, for instance, it is often assumed that agricultural productivity is the same for all croplands. The consideration of biophysical heterogeneity in ecosystems is particularly important for transferring values of habitat service, where ecological indicators contribute with 90% to the value transfer function. By utilizing more than 93 covariates we sought after the best possible solution to minimize uniformity error.

Regionalization error encompasses extrapolation errors due to generalization from a local case study to a wider region [83]. All value transfer functions are prone to errors according to different dimensions of scales [84]. The spatial extent of the investigation area showed high variation in the explanatory power of our analysis (1–26%). Most of the ES, except erosion and soil fertility, indicate diminishing returns to the spatial extent for ES values. The monetary value of an additional hectare to a large area is of lower value than an additional hectare to a smaller area, as reported for wetlands [13]. Further, resolution of data sets (granularity) determined explanatory values [85]. This was particularly striking for land use/land cover types according to the History Database of the Global Environment (HYDE). In 8 out of 12 ES land use/land cover types change fundamentally depending on the level of resolution. For lower-resolution HYDE (10 arc min) the ES recreation, for instance, showed the highest values in tropical forests and grasslands, however, according to the finer-resolution of study site description from the case studies coastal wetlands, partly inland wetlands and freshwater areas are the most worthwhile land use/land cover types, which confirms several preceding publications [13, 28, 29]. Missing indicators are, for instance, those that reflect how the value for spatially confined resources is expected to decline with increasing distance to that resource. Transfer values without accounting for distance decay may result in overestimations [86].

The major achievement of this study is, first, to clearly distinguish between major sources of uncertainties and to quantify patterns in the influence of different study- and site-characteristics that affect value transfer functions and predicted ES values. Second, the study identifies regions for which sufficient knowledge on our natural capital is available, i.e. a statistically defensible benefit transfer model can be applied in combination with uncertainty values. Third, we provide global maps of the “white spots” on our knowledge on accounting natural capital and ES. This provides guidance for future analysis and concerted action on the assessment of natural capital and ES within the work program of IPBES [87] and the Aichi Biodiversity Targets [88]. Fourth, we developed a conceptual basis for the establishment of a standardized integration and reporting of uncertainties in benefit transfer.

The integration of the presented value transfer functions in a fully automated spatial benefit transfer tool, such as the Ecosystem Valuation Toolkit [89], would be a next exciting step. Predefined automatic processes would reduce the time effort for model building and improve the model performance by constantly updating the data basis with both unconsidered covariates and case studies.

## Supporting Information

S1 Fig Global spatial distribution of monetary estimates and uncertainties, part 2. This map is the completion of the Fig 4 in the main article and shows the remaining six ES that were not presented in detail. The bivariate maps illustrate the extrapolated relative monetary values (yellow to green) and uncertainties (yellow to red) of the meta-analytic value transfer functions for the ES raw material provision (G), provision of medicinal resources(H), waste treatment (I), erosion regulation (J), soil fertility regulation (K) and Maintenance of genetic diversity (L).
(TIF)

S2 Fig Effects of most influential variables for twelve ES value transfer functions. Treemaps A) to L) represent models with covariates greater than 1%, their relative influence (or importance) and response. Rectangle groups delimited by bold black lines and numbered from 1 to 7 reflect groups of covariates mentioned in the main article (1) scale, 2) economy, 3) policy, 4) society, 5) ecology, 6) valuation methods, 7) rest <1%). The sizes of rectangles show the relative influence of covariates on a BRT model, in percentage. Rectangle colours illustrate the strength of relationship between monetary values and covariates. Greenish colours symbolize positive correlation and reddish negative, expressed in International-$-2007 per hectare. Multiple colours can occur for nonlinear effects of variables. Bluish colours represent categorical variables and show the maximum range between the levels.
(TIF)

S3 Fig Sensitivity analysis of BRT models. The graphs show the model performance for twelve parameter configurations. In the table below these configurations are specified. In addition to learning rate (lr), number of trees (nt) and minimal number of observations in the terminal nodes (mintn), there is also the selected model visualized. The selected model represents the final BRT model used for value transfer. It reduces the deviance of residuals in the model (squared error loss) the most and thus explains the variance of monetary values best.
(TIF)

S1 Table Case studies included for value transfer functions. The table gives an overview of the case study references that were included for the boosted regression trees.
(PDF)

S2 Table Covariates included for value transfer functions. The table shows sources of covariates either from valuation databases [13, 36] or from other global datasets. Information is listed for the groups of covariates (1) scale, 2) economy, 3) policy/governance, 4) society, 5) ecology, 6) valuation methods) and the names, spatial and temporal scale as well as source of covariates. Only covariates are mentioned that show a statistical influence in one of the value transfer functions.
(PDF)

S1 Text Extended description for model fitting and uncertainties. (PDF)

S2 Text The reference list links citations used in Fig 5. (PDF)
