# Supplementary figures and images for: Uncertainty of Monetary Valued Ecosystem Services – Value Transfer Functions for Global Mapping
Source: PLoS One. 2016 Mar 3;11(3):e0148524. doi: 10.1371/journal.pone.0148524 (PMC4777407; doi:10.1371/journal.pone.0148524)

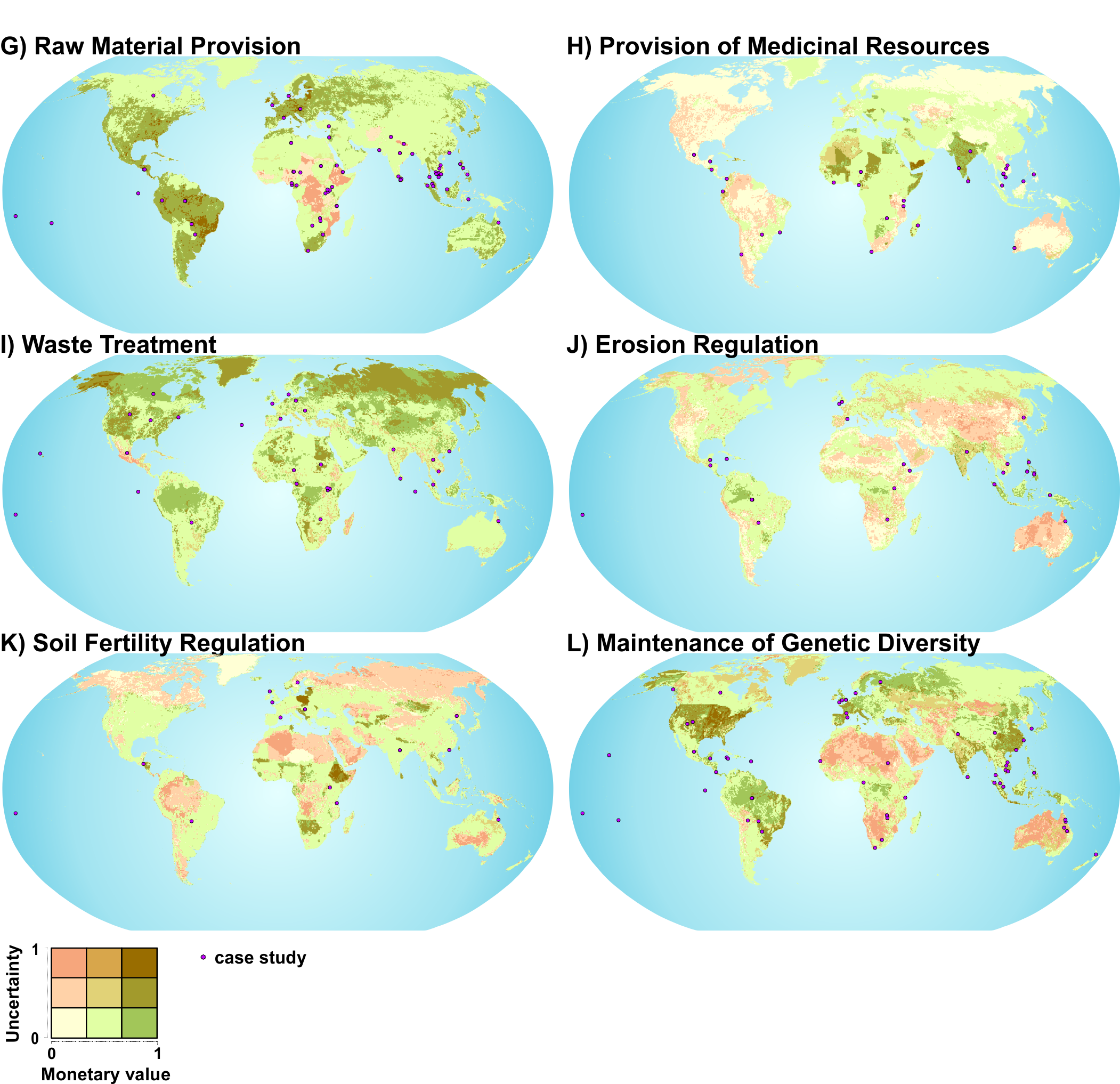

Supplement: S1 Fig — This map is the completion of the Fig 4 in the main article and shows the remaining six ES that were not presented in detail. The bivariate maps illustrate the extrapolated relative monetary values (yellow to green) and uncertainties (yellow to red) of the meta-analytic value transfer functions for the ES raw material provision (G), provision of medicinal resources(H), waste treatment (I), erosion regulation (J), soil fertility regulation (K) and Maintenance of genetic diversity (L). (TIF) [file pone.0148524.s001.tif]

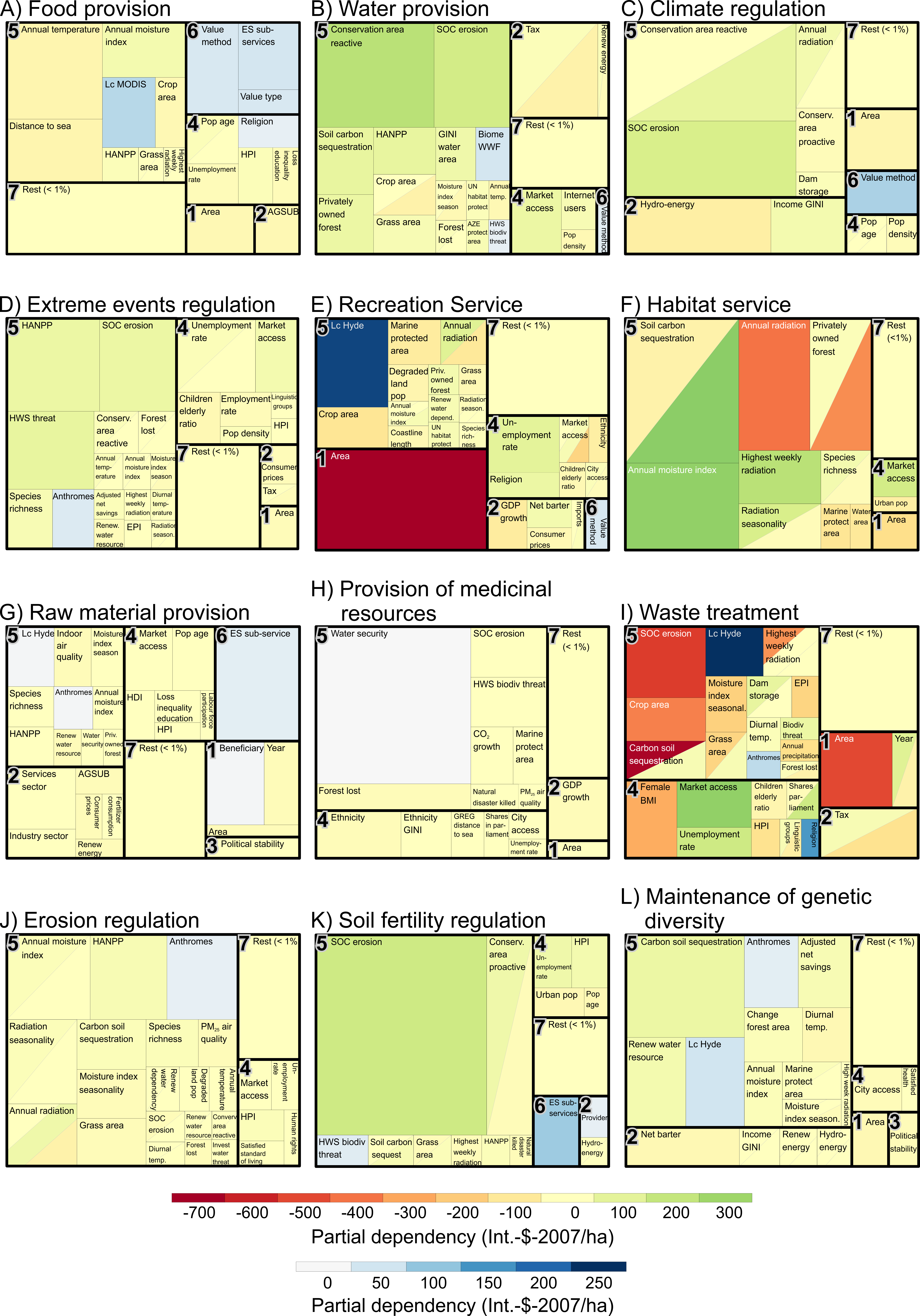

Supplement: S2 Fig — Treemaps A) to L) represent models with covariates greater than 1%, their relative influence (or importance) and response. Rectangle groups delimited by bold black lines and numbered from 1 to 7 reflect groups of covariates mentioned in the main article (1) scale, 2) economy, 3) policy, 4) society, 5) ecology, 6) valuation methods, 7) rest <1%). The sizes of rectangles show the relative influence of covariates on a BRT model, in percentage. Rectangle colours illustrate the strength of relationship between monetary values and covariates. Greenish colours symbolize positive correlation and reddish negative, expressed in International-$-2007 per hectare. Multiple colours can occur for nonlinear effects of variables. Bluish colours represent categorical variables and show the maximum range between the levels. (TIF) [file pone.0148524.s002.tif]

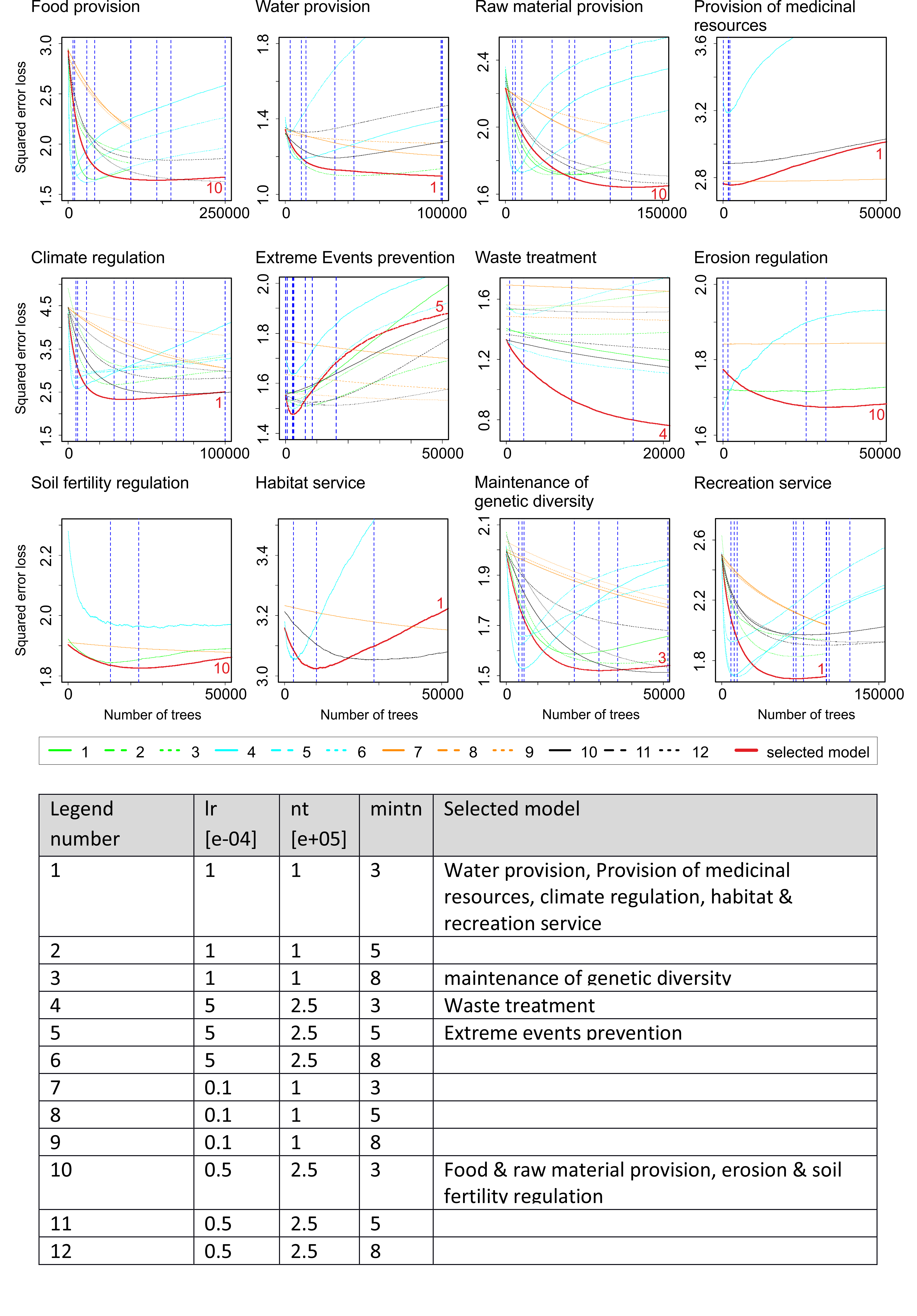

Supplement: S3 Fig — The graphs show the model performance for twelve parameter configurations. In the table below these configurations are specified. In addition to learning rate (lr), number of trees (nt) and minimal number of observations in the terminal nodes (mintn), there is also the selected model visualized. The selected model represents the final BRT model used for value transfer. It reduces the deviance of residuals in the model (squared error loss) the most and thus explains the variance of monetary values best. (TIF) [file pone.0148524.s003.tif]
