# Supplementary material for: Uncertainty of Monetary Valued Ecosystem Services – Value Transfer Functions for Global Mapping
Source: PLoS One. 2016 Mar 3;11(3):e0148524. doi: 10.1371/journal.pone.0148524 (PMC4777407; doi:10.1371/journal.pone.0148524)
Supplement: S1 Table — The table gives an overview of the case study references that were included for the boosted regression trees. (PDF) [file pone.0148524.s004.pdf]

S1 Table. Case studies included for value transfer functions. The table gives an overview of the case study references that were included for the boosted regression trees.

ID	Author	Year	Reference
1	Bann, C.	1999	A contingent valuation of the mangroves of Benut, Johor State, Malaysia. Report to DANCED, Copenhagen, Denmark.
2	Bostedt, G. and L. Mattsson	2006	A note on benefits and costs of adjusting forestry to meet recreational demands. Journal of Forest Economics 12(1): 75-81.
3	Loomis, J. and E. Ekstrand	1998	Alternative approaches for incorporating respondent uncertainty when estimating willingness-to-pay: The case of the Mexican spotted owl. Ecological Economics 27(1): 29-41.
4	Brenner, J., A. Jimenez, R. Sarda and A. Garola	2010	An assessment of the non-market value of the ecosystem services provided by the Catalan coastal zone, Spain.
5	Bann, C.	1997	An economic analysis of alternative mangrove management strategies in Koh Kong Province, Cambodia. Economy and Environment Program for Southeast Asia (EEPSEA research report series), International Development Research Centre.
6	Pyo, H.D.	2001	An economic analysis of preservation versus development of coastal wetlands around the Youngsan River. Ocean Policy Research 16
7	Bann, C.	1997	An economic analysis of tropical forest land use options, Ratanakiri Province, Cambodia. Economy and Environment Program for Southeast Asia, International Development Research Centre, Ottawa, Canada.
8	Thibodeau, F.R. and B.D. Ostro	1981	An economic analysis of wetland protection. Journal of Environmental Management 12: 19-30.
9	Gerrans, P.	1994	An economic valuation of the Jandakot wetlands. Western Australia: Edith Cowan University, ISBN: 0729801756. 100pp.
10	Do, T.N. and J. Bennett	2005	An economic valuation of wetlands in Vietnam's Mekong Delta: a case study of direct use values in Camau Province. Occasional Paper No. 8. Environment Management and Development Program, APSEG, ANU.
11	Luisetti, T., R.K. Turner and I.J. Bateman	2008	An ecosystem services approach to assess managed realignment coastal policy in England. CSERGE Working Paper ECM 08-04, CSERGE, University of East Anglia, Norwich, UK.
12	Hussain, S.S., A. Winrow-Giffin, D. Moran, L.A. Robinson, A. Fofana, O.A.L. Paramor and C.L.J. Frid	2010	An ex ante ecological economic assessment of the benefits arising from marine protected areas designation in the UK. Ecological Economics 69: 828-838.
13	Kasthala, G., A. Hepelwa, H. Hamiss, E. Kwayu, L. Emerton, O. Springate-Baginski, D. Allen, and W. Darwall	2008	An integrated assessment of the biodiversity, livelihood and economic value of wetlands in Mtanza-Msona Village, Tanzania. Tanzania Country Office, International Union for Conservation of Nature, Dar es Salaam.

14	Nam, P.K., and T.V.H. Son	2001	Analysis of the recreational value of the coral-surrounded Hon Mun slands in Vietnam. Environmental Economics Unit, Faculty of Development Economics, University of Economics, Vietnam.
15	Raboteur, J. and M.F. Rhodes	2006	Application de la méthode d'evaluation contingente aux récifs coralliens dans la Caraibe: etude appliquée a la zone de pigeon de la Guadeloupe. La revue électronique en sciences de l’environnement VertigO 7(1): 1-17.
16	De la Cruz, A. and J. Benedicto	2009	Assessing Socio-economic Benefits of Natura 2000: a Case Study on the ecosystem service provided by SPA PICO DA VARA / RIBEIRA DO GUILHERME. Output of the project Financing Natura 2000: Cost estimate and benefits of Natura 2000.
17	Emerton, L. and L.D.C.B. Kekulandala	2003	Assessment of the economic value of Muthurajawela Wetland. Working Paper. IUCN, Sir Lanka, 28pp.
18	Karanja, F., L. Emerton, J. Mafumbo and W. Kakuru	2001	Assessment of the economic value of pallisa district wetlands, Uganda. Biodiversity Economics for Eastern Africa & Uganda's National Wetlands Programme, IUCN Eastern Africa Programme.
19	Emerton, L.	1999	Balancing the opportunity costs of wildlife conservation for communities around Lake Mburo National Park, Uganda. Working paper, Institute for Development Policy and Management, University of Manchester, UK.
20	Rosales, R.M.P., M.F. Kallesoe, P. Gerrard, P. Muangchanh, S. Phomtavong and S. Khamsomphou	2005	Balancing the returns to catchment management. IUCN Water, Nature and Economics Technical Paper 5, IUCN, ecosystems and livelihoods group Asia, Colombo.
21	Zandersen, M., M. Termansen and F.S. Jensen	2005	Benefit transfer over time of ecosystem values: the case of forest recreation. FNU-61, Hamburg University and Centre for Marine and Atmospheric Science, Hamburg.
22	White, A.T., M. Ross and M. Flores	2000	Benefits and costs of coral reef and wetland management, Olango Island, Philippines. In: Cesar, H. (ed), "Collected essays on the economics of coral reefs". Kalmar, Sweden: CORDIO, Kalmar University: 215-227.
23	Naidoo, R. and W.L. Adamowicz	2005	Biodiversity and Nature-Based Tourism at Forest Reserves in Uganda, Environment and Development Economics 10(2): 158-178.
24	Islam, M. and J.B. Braden	2006	Bio-economic development of floodplains: farming versus fishing in Bangladesh. Environment and Development Economics 11: 95-126.
25	Emerton, L., R. Seilava and H. Pearith	2002	Bokor, Kirirom, Kep and Ream National Parks, Cambodia: Case Studies of Economic and Development Linkages. Field Study Report. International Centre for Environmental Management, Brisbane and IUCN.
26	Perrot-Maître, D. and P. Davis	2001	Case studies of markets and innovative financial mechanisms for water services from forests. Forest Trends, working paper.
27	Cooper, E., L. Burke and N. Bood	2009	Coastal capital : Belize - The economic contribution of Belize's coral reefs and mangroves. WRI Working Paper. World Resources Institute, Washington, D.C., 53pp.
28	Morton, R.M.	1990	Community structure, density, and standing crop of fishes in a subtropical Australian mangrove area. Marine Biology 105: 385-394.

29	Mallawaarachchi, T., R.K. Blamey, M.D. Morrison, A.K.L. Johnson and J.W. Bennet	2001	Community values for environmental protection in a cane farming catchment in Northern Australia: a choice modelling study. Journal of Environmental Management 62(3): 301-316.
30	Lal, P.N.	1990	Conservation or conversion of mangroves in Fiji. East-West Centre Occasional Papers 11
31	Regmi, B.N.	2003	Contribution of agroforestry for rural livelihoods: a case of Dhading District, Nepal. Paper presented at The International Conference on Rural livelihoods, Forests and Biodiversity 19-23 May 2003, Bonn, Germany.
32	Dubgaard, A., M.F. Kallesøe, M.L. Petersen and J. Ladenburg	2002	Cost-benefit analysis of the Skjern River Project. Royal veterinary and agricultural university. Conducted for the Danish Forest and Nature Agency as part of the investigations on biodiversity and nature protection by the Wilhjelm Committee.
33	Kniivila, M., V. Ovaskainen and O. Saastamoinen	2002	Costs and benefits of forest conservation: regional and local comparisons in Eastern Finland. Journal of Forest Economics 8(2): 131-150.
34	Anielski, M. and S.J. Wilson	2005	Counting Canada's natural capital: assessing the real value of Canada's boreal ecosystems. Canadian Boreal initiative, Pembina institute, Canadian.
35	Erdmann, M.V., P.R. Merrill, I. Arsyad and M. Mongdong	2003	Developing a diversified portfolio of sustainable financing options for Bunaken National Marine Park. Paper presented at 5th World Parks Congress: Sustainable Finance Stream, 2003. Durban, SA.
36	Mmopelwa, G., J.N. Blignaut and R. Hassan	2009	Direct use values of selected vegetation resources in the Okavango Delta Wetland. South African Journal of Economic and Management Sciences 12(2): 242-255.
37	Emerton, L.	1998	Djibouti biodiversity - economic assessment. IUCN, Gland, Switzerland.
38	Barbier, E.B., I. Strand and S. Sathirathai	2002	Do open access Conditions affect the valuation of an externality? Estimating the welfare effects of Mangrove-Fishery Linkages in Thailand. Environmental and Resource Economics 21(4): 343-367.
39	Kramer, R.A., D.D. Richter, S. Pattanayak and N.P. Sharma	1997	Ecological and Economic Analysis of Watershed Protection in Eastern Madagascar. Journal of Environmental Management 49: 277-295.
40	Hughes, Z.	2006	Ecological and economic assessment of potential eelgrass expansion at Sucia Island, WA.
41	Butcher Partners Limited	2006	Economic benefits of water in Te Papanui Conservation Park. Inception Report.
42	Gupta, T.R. and J.H. Foster	1975	Economic criteria for freshwater wetland policy in Massachussetts. American Journal of Agricultural Economics 57(1): 40-45.
43	Dixon, J.A. and G. Hodgson	1988	Economic evaluation of coastal resources: The El Niño study. Tropical Coastal Area Management (August): 5-7.
44	Pinedo-Vasquez, M., D. Zarin and P. Jipp	1992	Economic returns from forest conversion in the Peruvian Amazon. Ecological Economics 6(2): 163-173.
45	Cesar, H. and C.K. Chong	2004	Economic valuation and socioeconomics of coral feefs: methodological issues and three case studies. Wildfish Center Contribution No. 1721.
46	Gunawardena, M. and J.S. Rowan	2005	Economic valuation of a mangrove ecosystem threatened by shrimp aquaculture in Sri Lanka. Environmental Management 36(4): 535-550.

47	Hougner, C., J. Colding and T. Söderqvist	2006	Economic valuation of a seed dispersal service in the Stockholm National Urban Park, Sweden. Ecological Economics 59: 364-374.
48	Verma, M.	2001	Economic valuation of Bhoj Wetland for sustainable use. Indian Institute of Forest Management, Bhopal, EERC Working Paper Series: WB-9.
49	Samonte-Tan, G.P.B., A. T. White, M. A. Tercero, J. Diviva, E. Tabara and C. Caballes	2007	Economic Valuation of Coastal and Marine Resources: Bohol Marine Triangle, Philippines. Costal Management 35(2): 319-338.
50	Arntzen, J.	1998	Economic valuation of communal rangelands in Botswana: a case study. IIED, London, UK.
51	Chong, C.K., M. Ahmed and H. Balasubramanian	2003	Economic valuation of coral reefs at the Caribbean: literature review and estimation using meta-analysis. Paper presented at the Second International Tropical Marine Ecosystems Management Symposium.
52	Seenprachawong, U.	2003	Economic valuation of coral reefs at the Phi Phi Islands, Thailand. International journal for Global Environmental Issues 3(1): 104-114.
53	Burke, L., S. Greenhalgh, D. Prager and E. Cooper	2008	Economic valuation of coral reefs in Tobago and St. Lucia. Final report. World Resources Institute, Wahington, D.C..
54	Bellu L.G. and V. Cistulli	1997	Economic valuation of forest recreation facilities in the Liguria Region (Italy). Working paper GEC 97-08, Centre for Social and Economic Research on the Globlal Environment, Norwich, UK. ISSN 0967-8875.
55	Verma, M.	2000	Economic valuation of forests of Himachal Pradesh. International Institute for Environmental Development, London, UK.
56	Ammour, T., N. Windervoxhel and G. Sencion	2000	Economic valuation of mangrove ecosystems and sub-tropical forests in Central America. In: Dore M. and R. Guevara (ed), "Sustainable Forest management and Global Climate Change". Edward Elgar Publishing, UK.
57	Spaninks, F. and P. Van Beukering	1997	Economic valuation of mangrove ecosystems: potential and limitations. Economics of Environment and Development (CREED) Working Paper Series No. 14, 54pp.
58	Sathirathai, S.	1998	Economic valuation of mangroves and the roles of local communities in the conservation of natural resources: case study of Surat Thani, South Thailand. Unpublished report, EEPSEA research report series, Singapore.
59	Lynne, G.D., P. Conroy, and F.J. Pochasta	1981	Economic valuation of marsh areas to marine production processes. Journal of Environmental Economics and Management 8(2): 175-186.
60	Dubgaard, A.	1998	Economic valuation of recreational benefits from Danish Forests. In: Dabbert, S., A. Dubgaard and M. Whitby (ed), "The economics of Landscapes and Wildlife Conservation". CAB International: 53-64.
61	Cesar, H. and P. van Beukering	2004	Economic valuation of the coral reefs of Hawaii. Pacific Science 58(2), 231-242
62	Cesar, H., P. van Beukering, S. Pintz and J. Dierking	2002	Economic valuation of the coral reefs of Hawaii. Report for NOAA. Cesar Environmental Economics Consulting. Arnhem, the Netherlands.

63	Turpie, J.K., B.J. Heydenrych and S.J. Lamberth	2003	Economic value of terrestrial and marine biodiversity in the Cape Floristic Region: implications for defining effective and socially optimal conservation strategies. Biol. Conservation 112: 233-251.
64	Turpie, J., B. Smith, L. Emerton and J. Barnes	1999	Economic value of the Zambezi Basin Wetlands. Zambezi Basin Wetlands conservation and resource utilization project. IUCN Regional Office for Southern Africa.
65	Bergstrom, J.C., J.R. Stoll, J.P. Titre and V.L. Wright	1990	Economic value of wetlands-based recreation. Ecological Economics 2: 129-147.
66	Gren, I.M., K.H. Groth and M. Sylven	1995	Economic values of Danube floodplains. Journal of Environmental Management 45(4): 333-345.
67	Tong, C., R.A. Feagin, J. Lu, X. Zhang, X. Zhu, W. Wang and W. He	2007	Ecosystem service values and restoration in the urban Sanyang wetland of Wenzhou, China. Ecological Economics 29(3): 249-258.
68	Everard, M. and S. Jevons	2010	Ecosystem services assessment of buffer zone installation on the upper Bristol Avon, Wiltshire. Environment Agency.
69	Berg, H., M.C. Ohman, S. Troeng and O. Linden	1998	Environmental economics of coral reef destruction in Sri Lanka. Ambio 27(8): 627-634.
70	Navrud, S. and E.D. Mungatana	1994	Environmental valuation in developing countries: The recreational value of wildlife viewing. Ecological Economics 11(2): 135-151.
71	Emerton, L. and A. Asrat	1998	Eritrea biodiversity - economic assessment. IUCN, Gland, Switzerland.
72	Homarus Ltd.	2007	Estimate of economic values of activities in proposed conservation zone in Lyme Bay. A report for the wildlife trusts.
73	Donaghy, P., S. Chambers and I. Layden	2007	Estimating the economic consequences of incorporating BMP and EMS in the development of an intensive irrigation property in central Queensland.
74	Gammage, S.	1998	Estimating the returns to mangrove conversion: sustainable management or short term gain? Environmental Economics Programme, Discussion Paper. Presented at a workshop on Mechanisms for Financing Wise Use of Wetlands Dakar, Senegal, 13 November 1998.
75	Hargreaves-Allen, V.	2004	Estimating the total economic value of coral reefs for residents of Sampela, a Bajau community in Wakatobi Marine National, Sulawesi. A case study. MSc Thesis, Imperical College of Science, Technology and Medecine, UK.
76	Ly, O.K., J.T. Bishop, D. Moran and M. Dansohho	2006	Estimating the Value of Ecotourism in the Djoudj National Bird Park in Senegal. IUCN, Gland, Switzerland, 34pp.
77	Yaron, G.	2001	Forest, plantation crops or small-scale agriculture? An economic analysis of alternative land use options in the Mount Cameroun Area, Journal of Environmental Planning and Management 44(1): 85-108.
78	Charles, M.	2005	Functions and socio-economic importance of coral reefs and lagoons and implications for sustainable management. MSC Thesis, Wageningen University, the Netherlands.

79	De Groot, R.	1992	Functions of nature: evaluation of nature in environmental planning, management, and decision making. Wolters-Noordhoff, Groningen, the Netherlands, 315pp.
80	Seidl, A.F. and A.S. Moraes	2000	Global valuation of ecosystem services: application to the Pantanal da Nhecolandia, Brazil. Ecological Economics 33(1): 1-6.
81	Phillips, S., R. Silverman and A. Gore	2008	Greater than zero: toward the total economic value of Alaska's National Forest wildlands. The Wilderness Society, Washington, D.C., USA.
82	Lant, C.L. and R.S. Roberts	1990	Greenbelts in the cornbelt: riparian wetlands, instrinsic values and market failure. Environment and Planning A 22(10): 1375-1388.
83	Hamilton, L.S. and S.C. Snedaker	1984	Handbook for mangrove area management. East-West Environment and Policy Institute (Honolulu), 123pp.
84	Croitoru, L.	2007	How much are Mediterranean forests worth? Forest Policy and Economics 9(5): 536-545.
85	Gerrard, P.	2004	Integrating wetland ecosystem values into urban planning: the case of That Luang Marsh, Vientiane, Lao PDR. IUCN and WWF.
86	Ruitenbeek, J. and C. Cartier	1999	Issues in applied coral reef biodiversity valuation: results for Montego Bay, Jamaica. World Bank Research Committee Project RPO# 682-22. World Bank, Washington, D.C., USA.
87	Verweij, P., M. Schouten, P. Van Beukering, J. Triana, K. Van der Leeuw and S. Hess	2009	Keeping the Amazon forests standing: a matter of values. Report for WWF Netherlands.
88	Barrow, E. and H. Mogaka	2007	Kenya’s drylands: wastelands or an undervalued national economic resource. IUCN, Nairobi, Kenya.
89	Kosz, M., B. Brezina and T. Madreiter	1992	Kosten-Nutzen analyse ausgewahlter varianten eines nationalparks Donau-Auen. Institute fur Finanzwissenschaft and Infrastrukturpolitik der Technischen Universitat Wien, Austria
90	Schuijt, K.	2002	Land and water use of wetlands in Africa: economic values of African Wetlands. Interim Reports. International Institute for Applied Systems Analysis, Laxenburg, Austria.
91	Viglizzo, E.F. and F.C. Frank	2006	Land-use options for Del Plata Basin in South America: Tradeoffs analysis based on ecosystem service provision. Ecological Economics 57(1): 140-151.
92	Godoy, R., H. Overman, J. Demmer, L. Apaza, E. Byron, D. Wilkie, A. Cubas, K. McSweeney and N. Brokaw	2002	Local financial benefits of rain forests: comparative evidence from Amerindian societies in Bolivia and Honduras. Ecological Economics 40(3): 397-409.
93	Rodriguez, L.C., U. Pascual and H.M. Niemeyer	2006	Local identification and valuation of ecosystem goods and services from Opuntia scrublands of Ayacucho, Peru. Ecological Economics 57(1): 30-44.
94	Burbridge, P.R. and Koesoebiono	1984	Management of mangrove exploitation in Indonesia. In: Soepadmo, E., A.N. Rao and D.J. Macintosh (ed), "Proceedings Asian Symposium on Mangrove Environment: Research and Management". Kuala Lumpur, 25-29 Aug. 1980. University of Malaya and UNESCO.

95	Naidoo, R. and T.H. Ricketts	2006	Mapping the economic costs and benefits of conservation. PLoS Biology 4(11): 2153-2164.
96	Emerton, L. and Y. Tessema	2001	Marine protected areas: the case of Kisite Marine National Park and Mpunguti Marine National Reserve, Kenya. IUCN Eastern Africa Regional Office, Nairobi, Kenya.
97	Van der Heide, C.M., J.C.J.M. van den Bergh, E.C. van Ierland and P.A.L.D. Nunes	2005	Measureing the economic value of two habitat defragmentation policy scenarios for the Veluwe, The Netherlands. FEEM Working paper.
98	Hodgson G. and J. Dixon	1988	Measuring economic losses due to sediment pollution: logging versus tourism and fisheries. Tropical Coastal Area Management 3(1): 5-8
99	Nunes, P. A.L.D., L. Rossetto, and A. de Blaeij	2004	Measuring the economic value of alternative clam fishing management practices in the Venice Lagoon: results from a conjoint valuation application. Journal of Marine Systems 51: 309-320
100	Chang, W.K., C.O. Shin, C.H. Koh and S.H. Yoo	2009	Measuring the environmental value of Saeng Island in Busan, Korea with allowing for zero values. KMI International Journal 1: 24-31.
101	Dixon, J.A., L.F. Scura and T. van 't Hof	1993	Meeting ecological and economic goals: Marine parks in the Caribbean. Ambio - Biodiversity: Ecology, Economics, Policy 22(2/3): 117-125.
102	Ruitenbeek, H.J. (1994)	1994	Modelling economy-ecology linkages in mangroves: Economic evidence for promoting conservation in Bintuni Bay, Indonesia. Ecological Economics 10(3): 233-247
103	Emerton, L.	1998	Mont Kenya: the economics of community conservation. Institute for Development Policy and Management, University of Manchester, UK.
104	Shultz, S., J. Pinazzo and M. Cifuentes	1998	Opportunities and limitations of contingent valuation surveys to determine national park entrance fees: evidence from Costa Rica. Environment and Development Economics 3: 131-149.
105	Ruitenbeek J., M. Ridgley, S. Dollar, and R. Huber	1999	Optimization of economic policies and investment projects using a fuzzy logic based cost effectiveness model of coral reef quality: empirical results for Montego Bay, Jamaica. Coral Reefs 18: 381-392.
106	Pagiola, S., P. Agostini, J. Gobbi, C. de Haan, M. Ibrahim, E. Murgueitio, E. Ramírez, M. Rosales and J.P. Ruíz	2004	Paying for biodiversity conservation services in agricultural landscapes. Final draft. Forthcoming as Environment Department Paper No.96.
107	Whittingham, E., J. Cambell and P. Townsley (ed)	2003	Poverty and reefs. Volume 2: Case studies. DFID-IMM-IOC/UNESCO, 260pp.
108	Ayob, A., S. Rawi, S.A. Ahmad, and A. Arzem	2000	Preferences for outdoor recreation: The case of Pulau Payar Visitors
109	Janssen, R. and J.E. Padilla	1999	Preservation or Conversion? Valuation and evaluation of a mangrove forest in the Philippines. Environmental and Resource Economics 14(3): 297-331.
110	Walpole, M.J., H.J. Goodwin and K.G.R. Ward	2001	Pricing policy for tourism in protected areas: lessons from Komodo National Park, Indonesia. Conservation Biology 15(1): 218-227.

111	Bell, F.W. and V.R. Leeworthy	1990	Recreational demand by tourists for saltwater beach days. Journal of Environmental Economics and Management 18(3): 189-205.
112	Burke, L., E. Selig and M. Spalding	2002	Reefs at risk in Southeast Asia. World Resources Institute, Washington, D.C., ISBN 1-56973-490-9.
113	Sundberg, S.	2004	Replacement costs as economic values of environmental change: A review and an application to Swedish sea trout habitats. Beijer International Institute of Ecological Economics, The Royal Swedish Academy of Sciences.
114	Asquitha, N.M., M.T. Vargasa and S. Wunderb	2008	Selling two environmental services: In-kind payments for bird habitat and watershed protection in Los Negros, Bolivia. Ecological Economics 65(4): 675-684.
115	Cowling, R.M., R. Costanza and S.I. Higgins	1997	Services supplied by South African fynbos ecosystems. In: Daily, G. (ed), "Ecosystem services: their nature and value". Island Press, Washington, D.C., USA.
116	Ruitenbeek, H.J.	1988	Social cost-benefit analysis of the Korup Project, Cameroon. WWF for Nature Publication, London, UK.
117	Aubanel, A.	1993	Socioeconomic values of coral reef ecosystems and of its resources: a case study of an oceanic island in the South Pacific (Moorea, Society Islands). Universety Michel de Montange, Bordeaux, France.
118	Ahmad, N.	1984	Some aspects of economic resources of Sundarban mangrove forest of Bangladesh.
119	Eade, J.D.O. and D. Moran	1996	Spatial economic valuation: benefits transfer using geographical information systems. Journal of Environmental Management 48(2): 97-110.
120	Kumari, K.	1996	Sustainable forest management: myth or reality? Exploring the prospects for Malaysia. Ambio 25(7): 459-467.
121	Fleischer, A and Y. Tsur	2004	The amenity value of agricultural landscape and rural-urban land allocation. Discussion Paper No. 5.04, The Center for Agricultural Economic Research, The Hebrew University of Jerusalem, Isreal.
122	Amigues, J.-P., C. Boulatoff (Broadhead), B. Desaigues, C. Gauthier and J.E. Keith	2002	The benefits and costs of riparian analysis habitat preservation: a willingness to accept/willingness to pay contingent valuation approach. Ecological Economics 43(1): 17-31.
123	High, C. and C.M. Shackleton	2000	The comparative value of wild and domestic plants in home gardens of a South African rural village. Agroforestry Systems 48(2): 141-156.
124	Edwards, S.F.	1991	The demand for Galapagos vacations: estimation and application to wilderness preservation. Coastal Management 19: 155-199.
125	Adekola, O., S. Moradet, R. de Groot and F. Grelot	2008	The economic and livelihood value of provisioning services of Ga-Mampa wetland, South Africa. In: 13th IWRA World Water congress, 1 - 4 September, 2008, Montpellier, France.
126	Acess Economics	2008	The economic contribution of GBRMP - Report 2006-2007. Access Economics PTY Ltd. For Great Barrier Reef Marine Park Authority, Australia.
127	McArthur, L.C. and J.W. Boland	2006	The economic contribution of seagrass to secondary production in South Australia. Ecological Modelling 196(1-2): 163-172.

128	Fleischer, A. and M. Sternberg	2006	The economic impact of global climate change on Mediterranean rangeland ecosystems: a Space-for-Time approach. Ecological Economics 59(3): 287-295.
129	Barnes, J.I.	2002	The economic returns to wildlife management in Southern Africa. In: Pearce, D., C. Pearce and C. Palmer (ed), "The valuing the environment in developing countries: case studies". Cheltenham, UK and Northampton, MA, USA.
130	Riopelle, J.M.	1995	The economic valuation of coral reefs : a case study of West Lombok, Indonesia
131	Spurgeon, J.P.G.	1992	The economic valuation of coral reefs. Marine Pollution Bulletin 24(11): 529-536.
132	Bell, F.W.	1997	The economic valuation of saltwater marsh supporting marine recreational fishing in the southeastern United States. Ecological Economics 21(3): 243-254.
133	Blackwell, B.D.	2006	The economic value of Australia’s natural coastal assets: some preliminary findings. Australian and New Zealand Society for Ecological Economics Conference Proceedings, Ecological Economics in Action, December 11-13, 2005, New Zealand.
134	Pearce, D.W. and D. Moran	1994	The economic value of biodiversity. In association with the Biodiversity Programme of IUCN - The World Conservation Union, Earthscan Publications Ltd, London.
135	Muniz-Miret N., R. Vamos, M. Hiraoka. F. Montagnini and R.O. Mendelsohn	1996	The economic value of managing the acai palm (Euterpe oleracea Mart.) in the floodplains of the Amazon estuary, Para, Brazil. Forest Ecology and Management 87(1-3): 163-173.
136	Emerton, L., N. Erdenesaikhan, B. de Veen, D. Tsogoo, L. Janchivdorj, P. Suvd, B. Enkhtsetseg, G. Gandolgor, Ch. Dorjsuren, D. Sainbayar and A. Enkhbaatar	2009	The economic value of the upper tuul ecosystem, Mongolia. World Bank, Washington, D.C..
137	Department of Conservation	2007	The economic values of Whangamarino Wetland. Department of Conservation, DOCDM-141075.
138	Johnston, R.J., G. Magnusson, M.J. Mazzotta and J.J. Opaluch	2002	The economics of wetland ecosystem restoration and mitigation: combining economic and ecological indicators to Prioritize Salt Marsh Restoration Actions. American Journal of Agricultural Economics 84: 1362-1370.
139	Montenegro, L.O., A.G. Diola and E.M. Remedio	2005	The environmental costs of coastal reclamation in Metro Cebu, Philippines.
140	Meyerhoff, J. and A. Dehnhardt	2004	The European Water Framework Directive and Economic Valuation of Wetlands: the restoration of floodplains along the river Elbe. Working Paper on Management in Environmental Planning.
141	Turpie, J.K.	2003	The existence value of biodiversity in South Africa: how interest, experience, knowledge, income and perceived level of threat influence local willingness to pay. Ecological Economics 46(1-2): 199-216.

142	Cruz, W., H.A. Francisco and Z.T. Conway	1988	The on-site and downstream costs of soil erosion in the Magat and Pantabangan watersheds. Journal of Philippine Development 26: 85-11.
143	Emerton, L., L. Iyango, P. Luwum and A. Malinga	1998	The present economic value of Nakivubo Urban Wetland, Uganda. National Wetlands Conservation and Management Programme; IUCN: Biodiversity economics for Eastern Africa.
144	Yeo, B.H.	2004	The recreational benefits of coral reefs: A case study of Pulau Payar Marine Park, Kedah, Malaysia. In: Ahmed, M., C.K. Chong and H. Cesar (ed), "Economic valuation and policy priorities for sustainable management of coral reefs". WorldFish Center.
145	Bystrom, O.	2000	The replacement value of wetlands in Sweden. Environmental and Resource Economics 16(4):347-362
146	Loth, P. (ed)	2004	The return of the water restoring the Waza Logone floodplain in Cameroon. IUCN, Gland, Switzerland and Cambridge, UK.
147	Mohd-Shahwahid, H.O. and R. McNally	2001	The Terrestrial and Marin Resources of Samoa. Universiti Putra Malaysia, Malaysia.
148	Torras, M.	2000	The total economic value of Amazonian deforestation, 1978-1993. Ecological Economics 33(2): 283-297.
149	Turpie, J.K.	2000	The use and value of natural resources of the Rufiji Floodplain and Delta, Tanzania. Rufiji Environmental Managemet Project, Technical report No. 17.
150	Bennett, E.L. and C.J. Reynolds	1993	The value of a mangrove area in Sarawak. Biodiversity and Conservation 2(4): 359-375.
151	Gundimeda H., S. Sanyal, R. Sinha and P. Sukhdev	2006	The value of biodiversity in India's forests. Monograph 4 - Green Accounting for Indian States and Union Territories Project. TERI Press, New Delhi, India.
152	Seyam, I.M., A.Y. Hoekstra, G.S. Ngabirano and H.H.G. Savenije	2001	The value of freshwater wetlands in the Zambezi basin. Value of Water Research Report Series No. 7, IHE Delft, The Netherlands.
153	Chopra, K.	1993	The value of non-timber forest products: an estimation for tropical deciduous forests in India. Economic Botany 47(3): 251-257.
154	King, S.E. and J.N. Lester	1995	The value of salt marsh as a sea defence. Marine Pollution Bulletin 30 (3): 180-189.
155	Kontoleon, A. and T. Swanson	2003	The willingness to pay for property rights for the giant panda: can a charismatic species be an instrument for nature conservation. Land Economics 79(4): 483-499.
156	GEF/UNDP/IMO	1999	Total economic valuation: coastal and marine resources in the Straits of Malacca.
157	Adger, N., K. Brown, R. Cervigni, and D. Moran	1994	Towards estimating total economic value of forests in Mexico. GEC 94-21, Centre for Social and Economic Research on the Global Environment, University of East Anglia and University College London, UK.
158	Nickerson, D.J.	1999	Trade-offs of mangrove area development in the Philippines. Ecological Economics 28 (2): 279-298.
159	Emerton, L. and E. Muramira	1999	Uganda biodiversity - economic assessment. Prepared with National Environment Management Authority, Kampala. IUCN, Gland, Switzerland.
160	Everard, M.	2009	Using science to create a better place: ecosystem services case studies. Better regulation science programme. Environment Agency.

161	MANR	2002	Valoracion economica del humedal barrancones. Proyecto Regional de Conservación de los Ecosistemas Costeros del Golfo de Fonseca –PROGOLF.
162	Costanza, R., S. C. Farber, and J. Maxwell	1989	Valuation and management of wetlands ecosystems. Ecological Economics 1(4): 335-361.
163	Echeverria, J., M. Hanrahan and R. Solorzano	1995	Valuation of non-priced amenities provided by the biological resources whithin the Monteverde Cloud Forest preserve, Costa Rica. Ecological Economics 13(1): 43-52.
164	Tri, N.H.	2002	Valuation of the mangrove ecosystem in Can Gio mangrove biosphere reserve, Vietnam. The Vietnam MAB National Committee, UNESCO / MAB.
165	Lescuyer, G.	2007	Valuation techniques applied to tropical forest environmental services: rationale, methods and outcomes. Paper presented at the West and Central Africa Tropical Forest Investment Forum2007; Accra, Ghana" CIRAD/CIFOR, Yaoundé, Cameroon.
166	Emerton, L. and E. Bos	2004	Value: counting ecosystems as water infrastructure. IUCN, Gland, Switzerland.
167	Emerton, L (ed)	2005	Values and rewards: counting and capturing ecosystem water services for sustainable development. IUCN Water, Nature and Economics Technical Paper No. 1, IUCN — The World Conservation Union, Ecosystems and Livelihoods Group Asia.
168	Pendleton, L.H.	1995	Valuing coral reef protection. Ocean & Coastal Management 26(2): 119-131.
169	Carr, L. and R. Mendelsohn	2003	Valuing coral reefs: a travel cost analysis of the Great Barrier Reef. Ambio 32(5): 353-357.
170	Xue, D. and C. Tisdell	2001	Valuing ecological functions of biodiversity in Changbaishan Mountain Biosphere Reserve in Northeast China. Biodiversity and Conservation 10(3): 467-481.
171	Badola, R.and S.A. Hussain	2005	Valuing ecosystem functions: an empirical study on the storm protection function of Bhitarkanika mangrove ecosystem, India. Environmental conservation 32(1): 85-92.
172	Curtis, I.A.	2004	Valuing ecosystem goods and services: a new approach using a surrogate market and the combination of a multiple criteria analysis and a Delphi Panel to assign weights to the attributes. Ecological Economics 50: 163-194.
173	Barbier, E.B.	2007	Valuing ecosystem services as productive inputs. Economic Policy 22(1): 177-229.
174	Tobias D. and R. Mendelsohn	1991	Valuing ecotourism in a tropical rain-forest reserve. Ambio 20(2): 91-93.
175	Maille, P. and R. Mendelsohn	1993	Valuing ecotourism in Madagascar. Joural of Environmental Management 38: 213-218.
176	Kaiser, B. and J. Roumasset	2002	Valuing indirect ecosystem services: the case of tropical watersheds. Environment and Development Economics 7: 701-714.
177	Barbier, E.B.and I. Strand	1998	Valuing mangrove fishery linkages : a case study of Campeche, Mexico. Environmental and Resource Economics 12(2): 151-166.
178	Mathieu, L.F., I.H. Langford, W. Kenyon	2003	Valuing marine parks in a developing country: a case study of the Seychelles. Environment and Development Economics 8(2): 373-390.

179	Turner, R.K., J. Paavola, P. Cooper, S. Farber, V. Jessamy and S. Georgious	2003	Valuing nature: lessons learned and future research directions. Ecological Economics 46(3): 493-510.
180	Walsh, R.G., J.B. Loomis and R.A. Gillman	1984	Valuing option, existence, and bequest demand for wilderness. Land Economics 60(1): 14-29.
181	Ahmed, M., G.M. Umalia, C.K. Chong, M.F. Rull and M.C. Garcia	2007	Valuing recreational and conservation benefits of coral reefs: the case of Bolinao, Philippines. Ocean & Coastal Management 50(2): 103-118.
182	Blamey, R., J. Rolfe, J. Bennett and M. Morrison	2000	Valuing remnant vegetation in Central Queensland using choice modelling. The Australian Journal of Agricultural and Resource Economics 44(3): 439-456.
183	Rausser, G.C. and A.A. Small	2000	Valuing research leads: bioprospecting and the conservation of genetic resources. UC Berkeley: Berkeley Program in Law and Economics. Journal of Political Economy 108(1): 173-206.
184	Kosz, M.	1996	Valuing riverside wetlands: the case of the "Donau-Auen" national park. Ecological Economics 16: 109-127.
185	Croitoru, L.	2007	Valuing the non-timber forest products in the Mediterranean region. Ecological Economics 63(4): 768-775.
186	Grimes, A., S. Loomis, P. Jahnige, M. Burnham, K. Onthank, R. Alarcon, W.P. Cuenca, C.C. Martinez, D. Neil, M. Balick, B. Bennett and R. Mendelsohn	1994	Valuing the rain forest: the economic value of nontimber forest products in Ecuador. Ambio 23(7): 405-410.
187	Scarpa, R., S.M. Chilton, W.G. Hutchinson and J. Buongiorno	2000	Valuing the recreational benefits from the Creation of Natre Reserves in Irish forests. Ecological Economics 33(2): 237-250.
188	Chong, J.	2005	Valuing the role of aquatic resources in Livelihoods: economic aspects of community wetland management in Stoeng Treng Ramsar Site, Cambodia. IUCN Water, Nature and Economics Technical Paper No. 3.
189	Kramer, R.A., N.P. Sharma and M. Munashinghe	1995	Valuing tropical forests: Methodology and case study of Madagascar. World Bank Environment Paper 13.
190	Li, T., W. Li and Z. Qian	2008	Variations in ecosystem service value in response to land use changes in Shenzhen. Ecological Economics (In Press), Corrected Proof: 9.
191	Farber, S.	1996	Welfare loss of wetlands disintegration: a Louisiana study. Contemporary Economic Policy 14: 92-106
192	Dugan, P.J. (ed)	1990	Wetland conservation: a review of current issues and required action. IUCN, Gland, Switzerland.
193	Coreil, P.D.	1993	Wetlands functions and values in Louisiana. Louisiana Sea Grant publication, USA
194	Predo, C.D.	2003	What motivates farmers? Tree growing and land use ecisions in the grasslands of Claveria, Philippines. Research Report No. 2003-RR7, Economy an Environment Program for Southeast Asia (EEPSEA), Singapore.
