# Supplementary material for: Uncertainty of Monetary Valued Ecosystem Services – Value Transfer Functions for Global Mapping
Source: PLoS One. 2016 Mar 3;11(3):e0148524. doi: 10.1371/journal.pone.0148524 (PMC4777407; doi:10.1371/journal.pone.0148524)
Supplement: S2 Table — The table shows sources of covariates either from valuation databases [13, 36] or from other global datasets. Information is listed for the groups of covariates (1) scale, 2) economy, 3) policy/governance, 4) society, 5) ecology, 6) valuation methods) and the names, spatial and temporal scale as well as source of covariates. Only covariates are mentioned that show a statistical influence in one of the value transfer functions. (PDF) [file pone.0148524.s005.pdf]

S2 Table. Covariates included for value transfer functions. Sources of covariates either from valuation databases [13, 36] or from other global datasets. The table shows the groups of covariates (1) scale, 2) economy, 3) policy/governance, 4) society, 5) ecology, 6) valuation methods) and the names, spatial and temporal scale as well as source of covariates. Only covariates are listed that show a statistical influence in one of the value transfer functions.

Group	Name of variable	Unit	Spatial scale	Temporal scale	Source
1	Area<br>Spatial extent of the investigation area applied in the case study for each monetary valued ES.	Square kilometer	Site scale to continental	1975-2012	[13, 36]
1	Year<br>Publication year of the case study.	Year	Site scale to continental	1975-2012	[13, 36]
1	Beneficiary<br>Indication of population that benefits from enjoyment, consumption or use of valued ES.	Unitless index	Site scale to continental	1975-2012	[13, 36]
2	Provider<br>Type of supplier of ES or landowner of service area that is being valued, e.g. private, Non-Governmental Organization, Non-Profit Organization or government.	Unitless index	Local to international	1975-2012	[13, 36]
6	ES sub-services<br>Determined ES sub-classes based on the TEEB classification system. The TEEB categories consist of 27 ES, e.g. food provision or climate regulation, which were sub-divided into 74 sub-classes.	ES sub-types	Site scale to continental	1975-2012	[13, 36]
6	Value method<br>Classification of techniques used in the case study to value ES in monetary terms.	Type of method	Site scale to continental	1975-2012	[13, 36]
6	Value Type<br>The type of monetary “output" value from the case study, determined by the aggregated value of the ES benefits provided in a given state.	Type of value	Site scale to continental	1975-2012	[13, 36]
2	Consumer prices<br>A consumer price index is constructed to measure price changes over time for a fixed set of consumer goods and services of constant quantity and characteristics, acquired, used or paid for by households.	Unitless index	National level	2007	International Labour Office database provided by ILO. Retrieved 01/07/2015, from: http://laborsta.ilo.org/applv8/data/c7e.html

2	GDP growth<br>Annual percentage growth rate of Gross Domestic Product (GDP) per capita based on constant local currency. GDP per capita is gross domestic product divided by midyear population. GDP is the sum of gross value added by all resident producers in the economy (plus any product taxes and minus any subsidies, not included in the value of the products) measured in purchaser's prices. It is calculated without making deductions for depreciation of fabricated assets or for depletion and degradation of natural resources.	Annual percentage of GDP per capita	National level	2007	World Development Indicators provided by The World Bank. Retrieved 01/07/2015, from: http://data.worldbank.org/indicator/NY.GDP.PCAP.KD.ZG
2	Tax revenue<br>Tax revenue refers to compulsory financial transfers to the central government for public purposes. Certain compulsory transfers such as fines, penalties, and most social security contributions are excluded. Refunds and corrections of erroneously collected tax revenue are treated as negative revenue.	Annual percentage of GDP	National level	2007	World Development Indicators provided by The World Bank. Retrieved 01/07/2015, from: http://data.worldbank.org/indicator/GC.TAX.TOTL.GD.ZS
2	Income GINI<br>The income GINI coefficient measures the deviation of the distribution of income or consumption expenditure among individuals or households within an economy from a perfectly equal distribution.	Unitless index	National level	2000-2010	Cross-National Socio-Economic and Religion Data, 2011, from Association of Religion Data Archive (ARDA). Retrieved 01/07/2015, from: http://www.thearda.com/Archive/Files/Descriptions/ECON11.asp
2	Imports of goods and services<br>Represents the value of all goods and other market services received from the rest of the world. It includes the value of merchandise, freight, insurance, transport, travel, royalties, license fees, and other services, such as communication, construction, financial, information, business, personal, and government services.	Annual percentage of GDP	National level	2007	World Development Indicators provided by The World Bank. Retrieved 01/07/2015, from: http://data.worldbank.org/indicator/NE.IMP.GNFS.ZS
2	Net barter<br>The index net barter terms of trade is calculated as the percentage ratio of the export unit value indexes to the import unit value indexes, measured relative to the base year 2000 (2000=100).	Unitless index	National level	2007	World Development Indicators provided by The World Bank. Retrieved 01/07/2015, from: http://data.worldbank.org/indicator/TT.PRI.MRCH.XD.WD

2	Industry sector, value added<br>It comprises value added in mining, manufacturing, construction, electricity, water, and gas. Value added is the net output of a sector after adding up all outputs and subtracting intermediate inputs. It is calculated without making deductions for depreciation of fabricated assets or depletion and degradation of natural resources. The origin of value added is determined by the International Standard Industrial Classification (ISIC).	Annual percentage of GDP	National level	2007	World Development Indicators provided by The World Bank, Retrieved 01/07/2015, from: http://data.worldbank.org/indicator/NV.IND.TOTL.ZS
2	Services sector, value added<br>The indicator include value added in wholesale and retail trade (including hotels and restaurants), transport, and government, financial, professional, and personal services such as education, health care, and real estate services. Also included are imputed bank service charges, import duties, and any statistical discrepancies noted by national compilers as well as discrepancies arising from rescaling. Value added is the net output of a sector after adding up all outputs and subtracting intermediate inputs. It is calculated without making deductions for depreciation of fabricated assets or depletion and degradation of natural resources. The industrial origin of value added is determined by the International Standard Industrial Classification (ISIC).	Annual percentage of GDP	National level	2007	World Development Indicators provided by The World Bank. Retrieved 01/07/2015, from: http://data.worldbank.org/indicator/NV.SRV.TETC.ZS
2	AGSUB<br>Agricultural subsidies, i.e. governmental, financial support for farmers and agribusiness, ranks countries based on the magnitude of received subsidies, with first place representing highest subsidies. The magnitude of agriculture subsidies is expressed in price of their product in the domestic market (plus any direct output subsidy) less its global price, expressed as a percentage of the global price (adjusted for transport costs and quality differences).	Rank	National level	2007	Environmental Performance Index downloads webpage, hosted by Yale University. Retrieved 01/07/2015, from: http://epi.yale.edu/downloads
2	Net agriculture production index<br>The index show the relative level of the aggregate volume of agricultural production for each year in comparison with the reference period 2004-2006 (=100). It is based on the sum of price-weighted quantities of different agricultural commodities produced (crops and livestock) after deductions of quantities used as seed and feed weighted in a similar manner.	Unitless index	National level	2007	Data-platform of the Statistic Division of Food and Agriculture Organization of the United States (FAOSTAT). Retrieved 01/07/2015, from: http://faostat.fao.org/site/612/default.aspx#ancor

2	Hydro-energy<br>Per capita production of electricity from hydroelectric power plants during the given year, counted in tonne of oil equivalent (toe).	Tonne of oil equivalent (toe)	National level	2007	World Development Indicators provided by The World Bank. Retrieved 01/07/2015, from: http://data.worldbank.org/indicator/EG.ELC.HYRO.KH
2	Renew energy<br>Percentage of total primary energy supply derived from renewable sources. The renewable electricity production includes biodiesel, biogasoline, other biogas, charcoal, geothermal, hydro, other liquid biofuels, sludge gas, solar-photovoltaics, solar-thermal, tide wave & ocean, as well as wind.	Annual percentage of total electricity production	National level	2007	Online data services provided by the International Energy Agency. Retrieved 01/07/2015, from: http://data.iea.org
2	UNRENEW energy<br>Percentage of total primary energy supply derived from renewable sources.	Annual percentage of total electricity production	National level	2007	Cross-National Socio-Economic and Religion Data, 2011, from Association of Religion Data Archive (ARDA). Retrieved 01/07/2015, from: http://www.thearda.com/Archive/Files/Descriptions/ECON11.asp
3	Political stability<br>Reflects perceptions of the likelihood that the government will be destabilized or overthrown by unconstitutional or violent means, including politically-motivated violence and terrorism.	Unitless index	National level	2007	Worldwide Governance Indicators, hosted by The World Bank Group. Retrieved 01/07/2015, from: http://info.worldbank.org/governance/wgi/index.aspx#home
3	Country<br>States and its national administrative boundaries based on the classification of the International Organization for Standardization (ISO 3166-1) and United Nations Statistics Division.	Nations	National level	2008	Erle, S., G. Rich and J. Walsh, 2005, Mapping Hacks. O'Reilly & Associates. Inc. Sebastopol, CA, USA. ISBN:0596007035. Retrieved 01/07/2015, from: http://www.mappinghacks.com/data/
4	Urban pop<br>Population in urban agglomerations of more than one million measured by the percentage of a country's population living in metropolitan areas that in 2000 had a population of more than one million people.	Annual percentage of total population	National level	2007	World Development Indicators provided by The World Bank. Retrieved 01/07/2015, from: http://data.worldbank.org/indicator/EN.URB.MCTY.TL.ZS
4	Pop density<br>Number of inhabitants per square kilometer of total area in a country.	Inhabitants/km^2	National level	2007	AQUASTAT database provided by Food and Agriculture Organization of the United Nations (FAO). Retrieved 01/07/2015, from: http://www.fao.org/nr/water/aquastat/data/query/index.html?lang=en

4	Labour force participation rate<br>Ratio of female to male of the working-age population (ages 15–64) that actively engages in the labour market, by either working or actively looking for work.	Annual percentage of female to male shares	National level	2007	Statistics of the Human Development Report provided by the United Nations Development Programme. Retrieved 01/07/2015, from: http://hdr.undp.org/en/data
4	Employment rate<br>Ratio of employment to population expressed in percentage of the total population ages 15-64.	Annual percentage of total population ages 15-64	National level	2008	Cross-National Socio-Economic and Religion Data, 2011, from Association of Religion Data Archive (ARDA). Retrieved 01/07/2015, from: http://www.thearda.com/Archive/Files/Descriptions/ECON11.asp
4	HDI<br>The Human Development Index (HDI) can be viewed as an index of "potential" human development. It is a composite statistic of life expectancy, education, and income indices.	Unitless index	National level	2007	Statistics of the Human Development Report provided by the United Nations Development Programme. Retrieved 01/07/2015, from: http://hdr.undp.org/en/data
4	Loss due to inequality in education<br>The loss in mean years of schooling due to inequality is the difference between two averages – the arithmetic mean which does not account for inequality and the geometric mean which does. The loss, expressed as a percentage, is the relative difference between the two.	Annual percentage of difference between arithmetic and geometric mean of years of schooling	National level	2011	Statistics of the Human Development Report provided by the United Nations Development Programme. Retrieved 01/07/2015, from: http://hdr.undp.org/en/data
4	Shares in parliament<br>Female-male ratio of seats held by a respective gender in a lower or single house or an upper house or senate, where relevant.	Annual percentage of female-male ratio	National level	2008	Statistics of the Human Development Report provided by the United Nations Development Programme. Retrieved 01/07/2015, from: http://hdr.undp.org/en/data
4	Children elderly ratio<br>This total dependency ratio is the ratio of the sum of the population aged 0-14 and 65+ to the population aged 15-64. Data are shown as the proportion of dependents per 100 adults.	Ratio times 100	National level	2005	UN data from the United Nations Population Division provided by Gapminder. Retrieved 01/07/2015, from: http://www.gapminder.org/data/
4	Female BMI<br>The mean Body Mass Index (BMI) of the female population, is a mean counted in kilogram per square meter that is calculated under the assumption that each country has the same age composition as the world population.	Kilogram per square meter	National level	2007	Dataset of risk factors from the Global Burden of Metabolic Risk Factors of Chronic Diseases Collaborating Group hosted by Imperial College London. Retrieved 01/07/2015, from: http://www1.imperial.ac.uk/publichealth/departments/ebs/projects/eresh/majidezzati/healthmetrics/metabolicriskfactors/

4	Pop age<br>The indicator represents the average population age per country. Data are shown for six major age groups weighted by absolute number of people per age group.	Unitless index	National level	2007	International Data Base 2013 of the U.S. Census Bureau. Retrieved 01/07/2015, from: https://www.census.gov/population/international/data/idb/region.php
4	Unemployment rate<br>Unemployment rate in a country. Data are shown in percentage of the labor force that is without jobs.	Annual percentage	National level	2007	Cross-National Socio-Economic and Religion Data, 2011, from Association of Religion Data Archive (ARDA). Retrieved 01/07/2015, from: http://www.thearda.com/Archive/Files/Descriptions/ECON11.asp
4	City access<br>Travel time to major cities using road and rail networks or shipping lanes on navigable rivers, lakes and oceans. The calculation based on raster grids that contain values on time required to travel across them. It accounts for the infrastructure (roads, rivers), terrain characteristics (slopes, land-cover) and political factors (national boundaries).	Minutes of travel time	10 arc-minutes	2009	Uchida, H. and A. Nelson, 2008, Agglomeration Index: Towards a New Measure of Urban Concentration. Background paper for the World Bank's World Development Report 2009. Retrieved 01/07/2015, from: http://forobs.jrc.ec.europa.eu/products/gam/
4	Internet users<br>Internet users are people with access to the worldwide network. Measured in total number of people per country.	Total number of users	National level	2007	World Development Indicators provided by The World Bank. Retrieved 01/07/2015, from: http://data.worldbank.org/indicator/IT.NET.USER
4	Market access<br>Accessibility to markets based on measurements of travel time to cities and major maritime ports as proxies for domestic and international markets. It rather accounts for the infrastructure (roads, rivers) and a number of terrain characteristics (slopes, land-cover) that impede access to the markets than for the absolute distance. Furthermore a uniform velocity was used to calculate the total travel time.	Unitless index	10 arc-minutes	2011	P.H. Verburg, E.C. Ellis, A. Letourneau, 2011, A global assessment of market accessibility and market influence for global environmental change studies Environmental Research Letters, 6 Article No: 034019, 12 pp. Retrieved 01/07/2015, from: http://www.ivm.vu.nl/en/Organisation/departments/spatial-analysis-decision-support/Market_Influence_Data/index.asp
4	Linguistic groups<br>Number of linguistic groups, based on the global dataset Ethnologue (digitilized version of the Atlas Narodov Mira).	Total number of linguistic groups	National level	2000	Alesina, A.F., S. Michalopoulos and E. Papaioannou, 2012, Ethnic Inequality, Retrieved 01/07/2015, from: http://dx.doi.org/10.2139/ssrn.2169485

4	Ethnicity GINI<br>Deviation of spatial distribution of ethnicity groups within a country from a perfectly equal distribution. Gini coefficients are calculated across ethnicity groups for each country. Data for homeland ethnicity based on the global dataset Geo-Referencing of Ethnic Groups (GREG).	Unitless index	National level	2000	Alesina, A.F., S. Michalopoulos and E. Papaioannou, 2012, Ethnic Inequality, Retrieved 01/07/2015, from: http://dx.doi.org/10.2139/ssrn.2169485
4	Ethnicity<br>Number of ethnic groups per country based on the global dataset Geo-Referencing of Ethnic Groups (GREG).	Total number of ethnic groups	National level	2000	Alesina, A.F., S. Michalopoulos and E. Papaioannou, 2012, Ethnic Inequality, Retrieved 01/07/2015, from: http://dx.doi.org/10.2139/ssrn.2169485
4	GREG distance to Sea<br>Spatial inequality of distance to sea coasts across ethnic homelands. Gini coefficients are calculated across ethnicity homelands for each country. As a prerequisite the geodesic distance from the centroid of each ethnic homeland to the nearest coastline is measured in 1000s of km's. Data for homeland ethnicity based on the global dataset Geo-Referencing of Ethnic Groups (GREG).	Unitless index	National level	2012	Alesina, A.F., S. Michalopoulos and E. Papaioannou, 2012, Ethnic Inequality, Retrieved 01/07/2015, from: http://dx.doi.org/10.2139/ssrn.2169485
4	Ethnicity GINI >1%<br>Deviation of spatial distribution of ethnicity groups within a country from a perfectly equal distribution. Gini coefficients are calculated across ethnicity groups for each country. The indicator of ethnic inequality exclude small ethnicities, defined as groups capturing less than 1% of the population, in the year 2000, in a country.	Unitless index	National level	2000	Alesina, A.F., S. Michalopoulos and E. Papaioannou, 2012, Ethnic Inequality, Retrieved 01/07/2015, from: http://dx.doi.org/10.2139/ssrn.2169485
4	Land quality<br>Spatial inequality indicator of the average land quality for cultivation across ethnic homelands per country. The GINI index is the product of two components capturing the climatic and soil suitability for farming. The spatial analysis frame for the inequality measures are ethnic homelands based on the global dataset Geo-Referencing of Ethnic Groups (GREG).	Unitless index	National level	2002	Alesina, A.F., S. Michalopoulos and E. Papaioannou, 2012, Ethnic Inequality, Retrieved 01/07/2015, from: http://dx.doi.org/10.2139/ssrn.2169485
4	Religion<br>Largest religion by proportion per country.	Unitless index	National level	2007	Cross-National Socio-Economic and Religion Data, 2011, from Association of Religion Data Archive (ARDA). Retrieved 01/07/2015, from: http://www.thearda.com/Archive/Files/Descriptions/ECON11.asp

4	Satisfied health<br>Percentage of population that are satisfied with their personal health, most recent measure during 2006-2009.	Percentage of countries population	National level	2006-2009	Cross-National Socio-Economic and Religion Data, 2011, from Association of Religion Data Archive (ARDA). Retrieved 01/07/2015, from: http://www.thearda.com/Archive/Files/Descriptions/ECON11.asp
4	Satisfied standard of living<br>Percentage of population that are satisfied with their standard of living, most recent measure during 2006-2009.	Percentage of countries population	National level	2006-2009	Cross-National Socio-Economic and Religion Data, 2011, from Association of Religion Data Archive (ARDA). Retrieved 01/07/2015, from: http://www.thearda.com/Archive/Files/Descriptions/ECON11.asp
4	HPI<br>The Happy Planet Index (HPI) represents to topics, human well-being and environmental impact. The index uses global data on life expectancy (inequality adjusted average life expectancy), ladder of life (experienced well-being) and ecological footprint.	Unitless index	National level	2007	Abdallah S., J. Michaelson, S. Shah, L. Stoll, N. Marks, 2012, The Happy Planet Index: 2012 Report. A global index of sustainable well-being (nef: London). Retrieved 01/07/2015, from: http://www.happyplanetindex.org/data/
4	Human rights<br>The Human Rights Violations Score shows the level of civil and political rights violation based on the human rights definition of the Office of the United Nations High Commissioner for Human Rights.	Unitless index	National level	2008	Cross-National Socio-Economic and Religion Data, 2011, from Association of Religion Data Archive (ARDA). Retrieved 01/07/2015, from: http://www.thearda.com/Archive/Files/Descriptions/ECON11.asp
5	Fertilizer consumption<br>Is the sum of fertilizer consumption of the following types of nutrients: nitrogen (N), phosphate (P_20_5), potash (K_20) and including complex fertilizers (NP, PK, NK and NPK).	Total number of nutrients	National level	2007	Data-platform of the Statistic Division of Food and Agriculture Organization of the United States (FAOSTAT). Retrieved 01/07/2015, from: http://faostat.fao.org/site/575/DesktopDefault.aspx?PageID=575#ancor
5	Cropland area<br>(Sub-) National crop area statistics based on data from the Statistic Division of the FAO. Data are allocated to grid cells according to land covers from satellite image (DISCover version 2 data) of 2000 A.D. for cropland.	Cropland area km^2 per grid cell	5 arc-minutes	2000	Klein, K., Goldewijk, A. Beusen, G. van Drecht, M. de Vos, 2011, The HYDE 3.1 spatially explicit database of human-induced global land-use change over the past 12,000 years, Global Ecology and Biogeography, 20, pp. 73–86
5	Pasture area<br>(Sub-) National crop area statistics based on data from the Statistic Division of the FAO. Data are allocated to grid cells according to satellite map of 2000 A.D. for pasture.	Pasture area km^2 per grid cell	5 arc-minutes	2000	Klein, K., Goldewijk, A. Beusen, G. van Drecht, M. de Vos, 2011, The HYDE 3.1 spatially explicit database of human-induced global land-use change over the past 12,000 years, Global Ecology and Biogeography, 20, pp. 73–86

5	Moisture index seasonality<br>The moisture index is a measure of relative soil moisture. The Moisture index seasonality is the coefficient of variation calculated by the standard deviation of the weekly rainfall and evaporation estimates expressed as a percentage of the annual mean of those estimates.	Coefficient of variation	10 arc-minutes	1951-2000 mixed with 1961-1990	Kriticos, D.J., B.L. Webber, A. Leriche, N. Ota, I. Macadam, J. Bathols, J.K. Scott, 2012, CliMond: global high-resolution historical and future scenario climate surfaces for bioclimatic modelling, Methods in Ecology and Evolution, 3, pp. 53–64. Retrieved 01/07/2015, from: https://www.climond.org/Resources.aspx
5	Annual temperature<br>The annual mean of all the weekly mean temperatures. Each weekly mean temperature is the mean of that week's maximum and minimum temperature over the whole year.	Celsius	10 arc-minutes	1961-1990	Kriticos, D.J., B.L. Webber, A. Leriche, N. Ota, I. Macadam, J. Bathols, J.K. Scott, 2012, CliMond: global high-resolution historical and future scenario climate surfaces for bioclimatic modelling, Methods in Ecology and Evolution, 3, pp. 53–64. Retrieved 01/07/2015, from: https://www.climond.org/Resources.aspx
5	Diurnal temperature<br>The Diurnal Temperature Range is the difference between that week's diurnal maximum and minimum temperature averaged for a year.	Celsius	10 arc-minutes	1961-1990	Kriticos, D.J., B.L. Webber, A. Leriche, N. Ota, I. Macadam, J. Bathols, J.K. Scott, 2012, CliMond: global high-resolution historical and future scenario climate surfaces for bioclimatic modelling, Methods in Ecology and Evolution, 3, pp. 53–64. Retrieved 01/07/2015, from: https://www.climond.org/Resources.aspx
5	Annual precipitation<br>The sum of all 12 monthly precipitation estimates per year.	Millimeter	10 arc-minutes	1951-2000 mixed with 1961-1990	Kriticos, D.J., B.L. Webber, A. Leriche, N. Ota, I. Macadam, J. Bathols, J.K. Scott, 2012, CliMond: global high-resolution historical and future scenario climate surfaces for bioclimatic modelling, Methods in Ecology and Evolution, 3, pp. 53–64. Retrieved 01/07/2015, from: https://www.climond.org/Resources.aspx
5	Annual radiation<br>Weekly solar radiation estimates averaged over the whole year.	Watt per square meter	10 arc-minutes	1961-1990	Kriticos, D.J., B.L. Webber, A. Leriche, N. Ota, I. Macadam, J. Bathols, J.K. Scott, 2012, CliMond: global high-resolution historical and future scenario climate surfaces for bioclimatic modelling, Methods in Ecology and Evolution, 3, pp. 53–64. Retrieved 01/07/2015, from: https://www.climond.org/Resources.aspx

5	Highest weekly radiation<br>The largest solar radiation estimate for all weeks of the year.	Watt per square meter	10 arc-minutes	1961-1990	Kriticos, D.J., B.L. Webber, A. Leriche, N. Ota, I. Macadam, J. Bathols, J.K. Scott, 2012, CliMond: global high-resolution historical and future scenario climate surfaces for bioclimatic modelling, Methods in Ecology and Evolution, 3, pp. 53–64. Retrieved 01/07/2015, from: https://www.climond.org/Resources.aspx
5	Radiation seasonality<br>The coefficient of variation calculated by the standard deviation of the weekly solar radiation estimates expressed as a percentage of the annual mean of those estimates.	Coefficient of variation	10 arc-minutes	1961-1990	Kriticos, D.J., B.L. Webber, A. Leriche, N. Ota, I. Macadam, J. Bathols, J.K. Scott, 2012, CliMond: global high-resolution historical and future scenario climate surfaces for bioclimatic modelling, Methods in Ecology and Evolution, 3, pp. 53–64. Retrieved 01/07/2015, from: https://www.climond.org/Resources.aspx
5	Annual moisture index<br>The moisture index is a measure of relative soil moisture. Annual moisture index represents the mean over the whole year of all the weekly moisture index values. The weekly moisture index values are calculated from the weekly precipitation and evaporation values obtained from the climate surfaces in conjunction with the soil type and maximum soil water availability values supplied by the user.	Unitless index	10 arc-minutes	1951-2000 mixed with 1961-1990	Kriticos, D.J., B.L. Webber, A. Leriche, N. Ota, I. Macadam, J. Bathols, J.K. Scott, 2012, CliMond: global high-resolution historical and future scenario climate surfaces for bioclimatic modelling, Methods in Ecology and Evolution, 3, pp. 53–64. Retrieved 01/07/2015, from: https://www.climond.org/Resources.aspx
5	CO_2 growth<br>Average annual growth in per capita carbon dioxide emission from 1970-2008. Human-originated carbon dioxide emissions stemming from the burning of fossil fuels, gas flaring and the production of cement, including carbon dioxide emitted by forest biomass through depletion of forest areas, divided by mid-year population.	Annual percentage of CO_2 emission growth	National level	1970-2008	World Bank, 2013, World Development Indicator 2013. Washington D.C. provided by Statistics of the Human Development Report of the United Nations Development Programme. Retrieved 01/07/2015, from: http://hdr.undp.org/en/data-explorer
5	Indoor air quality<br>Percentage of population using solid fuel as the primary cooking fuel.	Annual percentage of population	National level	2007	Smith K.R., S. Mehta, M. Maeusezahl-Feuz, 2004, Indoor air pollution from household use of solid fuels. In: Comparative Quantification of Health Risks: Global and Regional Burden of Disease Attributable to Selected Major Risk Factors. Geneva: World Health Organization, 1435-1493. Retrieved 01/07/2015, from: http://epi.yale.edu/downloads

5	PM25 air quality<br>Population-weighted exposure to fine particulates ($PM_{2.5}$) in micro-grams per cubic meter. These data are derived from a model that was parameterized by MODIS Aerosol Optical Depth (AOD) data. The model covered all areas south of 60 degree north latitude and north of 60 degree south latitude.	Microgram per cubic meter	National level	2007	Boys, B.L., R.V. Martin, A. van Donkelaar, R. MacDonell, N.C. Hsu, M.J. Cooper, R.M. Yantosca, Z. Lu, D.G. Streets, O. Zhang, S. Wang 2014 Fifteen-year global time series of satellite-derived fine particulate matter, Environ. Sci. Technol, 10.1021/es502113p. Retrieved 01/07/2015, from: http://epi.yale.edu/downloads
5	Carbon soil sequestration<br>Suitability of soil conditions for carbon soil sequestration derived from the raster version of the FAO/UNESCO soil map of the world (wdsmwgr).	Unitless index	5 arc-minutes	2000	Food and Agriculture Organization of the United Nations. FAO GEONETWORK. Suitability of Soil conditions for Soil Carbon Sequestration (GeoLayer). Retrieved 01/07/2015, from: http://data.fao.org/map?entryId=f8c27d50-88fd-11da-a88f-000d939bc5d8
5	Change forest area<br>Percentage change in area under forest cover from 1990-2008.	Percentage of forest area	National level	1990-2008	Data-platform of the Statistic Division of Food and Agriculture Organization of the United States (FAOSTAT). Retrieved 01/07/2015, from: http://faostat.fao.org/site/377/default.aspx#ancor
5	Forest lost<br>The loss of forest area owing to deforestation from either human or natural causes, such as forest fires. This indicator uses a baseline forest cover layer (forest cover fraction with a 30% forest cover threshold) to measure the area under forest cover in the year 2000. It then combines forest loss estimates from Landsat for the periods 2000-2005 and 2005-2010 to arrive at a total forest cover change amount for the decade. This total is then divided by the forest area estimate for 2000 to come up with a percent change in forest cover over the decade.	Annual percentage of forest area	National level	2000-2010	Matthew C.H., V.S. Stephen and P.V. Potapov 2010: Quantification of global gross forest cover loss PNAS 107 (19) 8650-8655, doi:10.1073/pnas.0912668107. Retrieved 01/07/2015, from: http://epi.yale.edu/downloads
5	Privately owned forest<br>Percentage of forest land that is owned by individuals, families, private co-operatives, corporations, industries, private religious and educational institutions, pension or investment funds, and other private institutions.	Annual percentage of forests	National level	2005	Rametsteiner E., A. Whiteman and E. Muller, 2014, State of the world's forests 2014. Enhancing the socioeconomic benefits from forests. FAO, Rome. Provided by Gapminder. Retrieved 01/07/2015, from: http://www.gapminder.org/data/

5	Biodiv threat<br>Riverine biodiversity threatened by stressors like catchment disturbance, pollution, water resource development and biotic factors. Calculated by using relative weights of 23 combined geospatial stressors to derive a cumulative threat index. Expert assessment of stressor impacts on biodiversity produced the weights.	Unitless index	30 arc-minutes	2010	Vorosmarty, C.J., P.B. McIntyre, M.O. Gessner, D. Dudgeon, A. Prusevich, P. Green, S. Glidden, S.E. Bunn, C.A. Sullivan, C. Reidy Liermann and P.M. Davies, 2010, Global threats to human water security and river biodiversity, Nature 467, 555-561. Retrieved 01/07/2015, from: http://www.riverthreat.net/data.html
5	Coastline length<br>Length of coastline in kilometers per country.	Kilometers	National level	2011	Cross-National Socio-Economic and Religion Data, 2011, from Association of Religion Data Archive (ARDA). Retrieved 01/07/2015, from: http://www.thearda.com/Archive/Files/Descriptions/ECON11.asp
5	Water area<br>Water area is the sum of the surfaces of all inland water bodies, such as lakes, reservoirs, or rivers, as delimited by international boundaries and/or coastlines.	Square kilometer	National level	2011	Cross-National Socio-Economic and Religion Data, 2011, from Association of Religion Data Archive (ARDA). Retrieved 01/07/2015, from: http://www.thearda.com/Archive/Files/Descriptions/ECON11.asp
5	Distance to Sea<br>Spatial inequality of the distance to sea across artificial pixels (of 2.5 x 2.5 decimal degrees). As a measure of the distance to sea the geodesic distance from the centroid of each pixel to the nearest coastline is estimated. Gini coefficients are calculated across these pixels for each country.	Unitless index	National level	2012	Global Mapping International, Colorado Springs, Colorado, USA. Series name: Global Ministry Mapping System. Series issue: Version 3.0
5	Water security<br>Degradation of freshwater resources threatened by stressors like catchment disturbance, pollution, water resource development and biotic factors. Human water security threat calculated by using relative weights of 23 combined geospatial stressors to derive a cumulative threat index. Expert assessment of stressor impacts on human water security produced the weights.	Unitless index	30 arc-minutes	2010	Vorosmarty, C.J., P.B. McIntyre, M.O. Gessner, D. Dudgeon, A. Prusevich, P. Green, S. Glidden, S.E. Bunn, C.A. Sullivan, C. Reidy Liermann and P.M. Davies, 2010, Global threats to human water security and river biodiversity, Nature 467, 555-561. Retrieved 01/07/2015, from: http://www.riverthreat.net/data.html

5	Invest water threat<br>Technological investments aimed at reducing threats to human water security. The investment factor depicting supply stabilization, improved water services and access to waterways.	Unitless index	30 arc-minutes	2010	Vorosmarty, C.J., P.B. McIntyre, M.O. Gessner, D. Dudgeon, A. Prusevich, P. Green, S. Glidden, S.E. Bunn, C.A. Sullivan, C. Reidy Liermann and P.M. Davies, 2010, Global threats to human water security and river biodiversity, Nature 467, 555-561. Retrieved 01/07/2015, from: http://www.riverthreat.net/data.html
5	HWS biodiv threat<br>Exposure to human water security (HWS) threat and biodiversity degradation. HWS is driven by stressors like catchment disturbance, pollution, water resource development and biotic factors (see water security). HWS threat is converted to reduced threat by capturing technological investments that can improve HWS and contrasted against incident biodiversity threat (see Biodiv prevail threat).	Unitless index	30 arc-minutes	2010	Vorosmarty, C.J., P.B. McIntyre, M.O. Gessner, D. Dudgeon, A. Prusevich, P. Green, S. Glidden, S.E. Bunn, C.A. Sullivan, C. Reidy Liermann and P.M. Davies, 2010, Global threats to human water security and river biodiversity, Nature 467, 555-561. Retrieved 01/07/2015, from: http://www.riverthreat.net/data.html
5	GINI water area<br>Spatial inequality of water area across artificial pixels (of 2.5 x 2.5 decimal degrees). Water areas are measured by the sum of the surfaces of all inland water bodies, such as lakes, reservoirs, or rivers in square kilometers, delimited by pixel boundaries and/or coastlines.	Unitless index	30 arc-minutes	2012	Global Mapping International, Colorado Springs, CO, USA. Global Ministry Mapping System. IN: Alesina, Alberto F. and Michalopoulos, Stelios and Papaioannou, Elias, Ethnic Inequality (October 31, 2012). Retrieved 01/07/2015, from: http://dx.doi.org/10.2139/ssrn.2169485
5	Renewable water resources<br>The sum of internal renewable water resources and external actual renewable water resources. It corresponds to the maximum theoretical yearly amount of water actually available for a country at a given moment. It was calculated by: [Water resources: total renewable (actual)] = [Surface water: total renewable (actual)] + [Groundwater: total renewable (actual)] - [Overlap between surface water and groundwater].	Cubic meter per year	National level	2007	AQUASTAT database provided by Food and Agriculture Organization of the United Nations (FAO). Retrieved 01/07/2015, from: http://www.fao.org/nr/water/aquastat/data/query/index.html
5	Dam storage<br>Total cumulative storage capacity of all dams in each country. The value indicates the sum of the theoretical initial capacities of all dams, which does not change with time.	Cubic meter	National level	2003-2007	AQUASTAT database provided by Food and Agriculture Organization of the United Nations (FAO). Retrieved 01/07/2015, from: http://www.fao.org/nr/water/aquastat/data/query/index.html

5	Renew water dependency<br>The Indicator expresses the percentage of total renewable water resources originating outside the country. The higher the indicator the more renewable water is received from neighboring countries such as upstream areas.	Annual percentage of total renewable water resources originating outside the country	National level	2007	AQUASTAT database provided by Food and Agriculture Organization of the United Nations (FAO). Retrieved 01/07/2015, from: http://www.fao.org/nr/water/aquastat/data/query/index.html
5	SOC erosion<br>The total erosion of soil organic carbon (SOC) by water and tillage on croplands. Global estimates based on a revised Universal Soil Loss Equation (USLE) model and are computed for a global grid of 5 arc-minutes.	Milligram Carbon per hectare and year	5 arc-minutes	2007	Van Oost, K., T.A. Quine, G. Govers, S. De Gryze, J. Six, J.W. Harden, J.C. Ritchie, G.W. McCarty, G. Heckrath, C. Kosmas, J.V. Giraldez, J.R.M. da Silva, R. Merckx. 2007. The impact of agricultural soil erosion on the global carbon cycle, Science, 318, pp. 626–629
5	Adjusted net savings<br>The indicator represents the true rate of savings in an economy after taking into account investments in human capital, depletion of natural resources and damage caused by pollution. Adjusted net savings are equal to net national savings plus education expenditure and minus energy depletion, mineral depletion, net forest depletion, and carbon dioxide and particulate emissions damage.	Percentage of GNI	National level	2007	World Development Indicators provided by The World Bank. Retrieved 01/07/2015, from: http://data.worldbank.org/indicator/NY.ADJ.SVNG.GN.ZS
5	Anthromes<br>Anthropogenic Biomes of the world (anthromes) indicate globally-significant ecological patterns within the terrestrial biosphere caused by human interaction with ecosystems, including agriculture, urbanization, forestry and other land uses.	Anthrome types	5 arc-minutes	2008	Ellis, E.C. and N. Ramankutty. 2008. Anthropogenic Biomes of the World, Version 1. Palisades, NY: NASA Socioeconomic Data and Applications Center (SEDAC). Retrieved 01/07/2015, from: http://sedac.ciesin.columbia.edu/data/set/anthromes-anthropogenic-biomes-world-v1
5	EPI<br>The Environmental performance Index (EPI) describes environmental compatibility of a state's policy concerning to human health affected by ecosystem health and human interventions, such as ecosystem protection and resource management. The EPI focused on a core set of environmental outcomes linked to policy goals.	Unitless index	National level	2010	Emerson, J., D. C. Esty, M.A. Levy, C.H. Kim, V. Mara, A. de Sherbinin, and T. Srebotnjak. 2010. 2010 Environmental Performance Index. New Haven: Yale Center for Environmental Law and Policy. Retrieved 01/07/2015, from: http://epi.yale.edu/files/2010_epi_report.pdf

5	HANPP<br>Human appropriation of net primary production (HANPP) is the human alteration of photosynthetic products in ecosystem and the harvest of those products. It represents the combined effect of harvest and productivity change induced by land use (e.g. land cover change, soil degradation) on the availability of Net Primary Production (NPP) in ecosystems. NPP is the amount of biomass produced each year by plants [kg C/yr].	Annual percentage of Net Primary Production	5 arc-minutes	2007	Haberl, H., K.H. Erb, F. Krausmann, V. Gaube, A. Bondeau, C. Plutzar, S. Gingrich, W. Lucht, M. Fischer-Kowalski. 2007. Quantifying and mapping the human appropriation of net primary production in earth's terrestrial ecosystems, Proceedings of the National Academy of Sciences of the United States of America, 104, pp. 12942–12945. Retrieved 01/07/2015, from: http://www.uni-klu.ac.at/socec/inhalt/1191.htm
5	Degraded land pop<br>Percentage of the population living on severely or very severely degraded land. Land degradation estimates consider biomass, soil health, water quantity and biodiversity.	Annual percentage of population per year	National level	2012	Statistics of the Human Development Report provided by the United Nations Development Programme. Retrieved 01/07/2015, from: https://data.undp.org/dataset/Population-living-on-degraded-land-/nric-gjtm
5	Lu/lc HYDE<br>Land use/land cover (lu/lc) data of the History Database of the Global Environment (HYDE). HYDE represents historical population and land use patterns for the past 300 years. For this analysis, however, only the most recent gridded maps of the lu/lc types were used.	Land use/land cover types	10 arc-minutes	1990	Klein Goldewijk, K. 2001. Estimating global land use change over the past 300 years: the HYDE database. Global Biogeochemical Cycles 15(2): 417-434. Retrieved 01/07/2015, from: http://themasites.pbl.nl/tridion/en/themasites/hyde/download/index-2.html
5	Lc MODIS<br>Global land cover (lc) dataset based on the Moderate Resolution Imaging Spectroradiometer (MODIS) Collection 5 Land Cover Type. Land cover classes (17-class scheme) are defined according to the International Geosphere-Biosphere Programme (IGBP).	Land cover types	0.5 arc minutes	2010	Friedl, M. A., D. Sulla-Menashe, B. Tan, A. Schneider, N. Ramankutty, A. Sibley and X. Huang, 2010; MODIS Collection 5 global land cover: Algorithm refinements and characterization of new datasets. Remote Sensing of Environment, 114, 168-182.
5	Biome WWF<br>Global land cover map consisting of bioclimatic zones that differ in climatic conditions and vegetation types. For the delineation of the land cover types a synthesis of previous biogeographic studies was conducted (see Olson et al. 2001). For this analysis the 16-class scheme of major biomes was used.	Land cover types	0.5 arc minutes	2001	Olson, D. M., E. Dinerstein, E.D. Wikramanayake, N.D. Burgess, G.V.N. Powell, E.C. Underwood, J.A. D'Amico, I. Itoua, H.E. Strand, J.C. Morrison, C.J. Loucks, T.F. Allnutt, T.H. Ricketts, Y. Kura, J.F. Lamoreux, W:W. Wettengel, P. Hedao, K.R. Kassem, 2001, Terrestrial ecoregions of the world: a new map of life on Earth. Bioscience 51(11):933-938. Retrieved 01/07/2015, from: https://worldwildlife.org/publications/terrestrial-ecoregions-of-the-world

5	AZE protection<br>Critical habitat protection sites that are identified by the Alliance of Zero Extinction (AZE). Each site represents the last refuge of one or more of the world's most highly threatened species. These sites are specified as percentage of the total AZE sites within each country that are within a protected area.	Percentage area of AZE site(s) that fell within protected areas per country	National level	2007	Ricketts, T.H., E. Dinerstein, T. Boucher, T.M. Brooks, S.H.M. Butchart, M. Hoffmann, J.F. Lamoreux, J. Morrison, M. Parr, J.D. Pilgrim, A.S.L. Rodrigues, W. Sechrest, G.E. Wallace, K. Berlin, J. Bielby, N.D. Burgess, D.R. Church, N. Cox, D. Knox, C. Loucks, G.W. Luck, L.L. Master, R. Moore, R. Naidoo, R. Ridgely, G.E. Schatz, G. Shire, H. Strand, W. Wettengel, and E. Wikramanayake, 2005, Pinpointing and preventing imminent extinctions PNAS 102 (51) 18497-18501. Retrieved 01/07/2015, from: http://epi.yale.edu/downloads
5	Marine Protected Area<br>The percentage of each country's Exclusive Economic Zone (EEZ, 0-200 nautical miles) that is under protection by a marine protected area (MPA). MPAs may include non-take marine reserves (forbidden: fishing, aquaculture, dredging, and mining, usually allowed: swimming, boating, and scuba diving), as well as other zones in which multiple use is afforded. The vast majority of MPAs, both in terms of numbers and area, are open for fishing, diving, boating, and other recreational and commercial uses.	Percentage of each country's Exclusive Economic Zone (EEZ)	National level	2011	Wood, L.J., 2007, MPA Global: A database of the world's marine protected areas. Sea Around Us Project, UNEP - WCMC & WWF. Retrieved 01/07/2015, from: http://epi.yale.edu/downloads
5	Conservation area proactive<br>Habitat and species protection areas that prioritizing low vulnerability, but high irreplaceability. These areas are characterized by low threat of biodiversity loss, but high uniqueness of habitat and/or species. The indicator is based on an overlay of different conservation approaches, such as species and habitat diversity, species and habitat rarity, ecological functions at risk etc., in order to see whether certain areas are consistently identified as being of high priority.	Unitless index	30 arc-minutes	2006	Brooks T.M., R.A. Mittermeier, G.A. da Fonseca, J. Gerlach, M. Hoffmann, J.F. Lamoreux, C.G. Mittermeier, J.D. Pilgrim, A.S. Rodrigues, 2006, Global biodiversity conservation priorities. Science 313, 58–61. doi:10.1126/science.1127609

5	Conservation area reactive<br>Areas that prioritizing high vulnerability of conservation areas in highly irreplaceable regions. These areas are characterized by a high threat that unique biodiversity will soon be lost unless immediate conservation action is taken within them. The indicator is based on an overlay of different conservation approaches, such as species and habitat diversity, species and habitat rarity, ecological functions at risk etc., in order to see whether certain areas are consistently identified as being of high priority.	Unitless index	30 arc-minutes	2006	Brooks T.M., R.A. Mittermeier, G.A. da Fonseca, J. Gerlach, M. Hoffmann, J.F. Lamoreux, C.G. Mittermeier, J.D. Pilgrim, A.S. Rodrigues, 2006, Global biodiversity conservation priorities. Science 313, 58–61. doi:10.1126/science.1127609
5	Species richness<br>Total number of species including taxonomic groups of terrestrial mammals, birds, reptiles and amphibians. The indicator is based on global range polygon data from the International Union for Conservation of Nature (IUCN) database.	Total number of species per grid cell	Calculated from range polygons	2014	IUCN, 2015, The IUCN Red List of Threatened Species. Spatial data download. Retrieved 01/07/2015, from: http://www.iucnredlist.org/technical-documents/spatial-data
5	UN habitat protected<br>Percentage of terrestrial area that is officially designated as protected by countries. Data is based on the United Nations List of Protected Areas.	Percentage of terrestrial area per nation	National level	2009	Cross-National Socio-Economic and Religion Data, 2011, from Association of Religion Data Archive (ARDA). Retrieved 01/07/2015, from: http://www.thearda.com/Archive/Files/Descriptions/ECON11.asp
5	Natural disasters killed<br>Number of people killed due to natural disasters such as floods, wildfires, mass movements etc. per country. For a disaster one of the following criteria must be fulfilled: Ten (10) or more people reported killed, hundred or more people reported affected, declaration of a state of emergency, call for international assistance.	Number of deaths average per year, per one million people	National level	2010	Human Development Indicator by country provided by United Nation Development Programme 2011, hosted by Socrata. Retrieved 01/07/2015, from: https://data.undp.org/dataset/HDI-Indicators-by-Country-2013/hah6-ygzy/widget_preview?height=425&variation=vb6g-9i38&width=500
5	Natural disasters affected<br>Number of people affected by natural disasters such as floods, wildfires, mass movements etc. per country. For a disaster one of the following criteria must be fulfilled: Ten (10) or more people reported killed, hundred or more people reported affected, declaration of a state of emergency, call for international assistance.	Average number per year, per one million people	National level	2000-2009	Cross-National Socio-Economic and Religion Data, 2011, from Association of Religion Data Archive (ARDA). Retrieved 01/07/2015, from: http://www.thearda.com/Archive/Files/Descriptions/ECON11.asp
