# Supplementary material for: Uncertainty of Monetary Valued Ecosystem Services – Value Transfer Functions for Global Mapping
Source: PLoS One. 2016 Mar 3;11(3):e0148524. doi: 10.1371/journal.pone.0148524 (PMC4777407; doi:10.1371/journal.pone.0148524)
Supplement: S1 Text — (PDF) [file pone.0148524.s006.pdf]

S1 Text. Extended description for model fitting and uncertainties

For the analysis the following assumptions have been made [26]:

(1) the existence of meta valuation functions from which values can be inferred;

(2) that differences between sites can be captured through a monetary vector [9];

(3) values are supposed to vary in a systematic way captured by a price deflator index [97];

(4) primary valuation studies provide “correct” estimates of marginal values; and

(5) meta-analytic publication selection errors can be neglected.

We created meta-analytic value transfer functions for each ES based on boosted regression trees (BRT). Therefore, we utilized the Generalized Boosted Regression Models library [41, 42] from the programming language R [43]. We normalized the response variable by using a log transformation and fitted the BRT models to a Gaussian response type. The relative influence (importance) of covariates in the BRT models were calculated based on the number of times a variable is selected for splitting, weighted by the squared improvement of the model as a result of each split, and averaged over all trees [98]. Then we verified the robustness and stability of the valuation functions by changing BRT model parameters. Model settings were derived from recommendations of most up-to-date literature. We tested different model settings by optimizing BRT learning rate (lr), tree complexity (tc), minimal number of observations in terminal nodes (mintn) and number of trees (nt).

The lr controls the rate model complexity is increased. Smaller lr are generally preferable to faster ones, because they shrink the contribution of each tree more, and help the model to reliably estimate the response [99]. We used values from 0.0005, 0.0001, 0.00005, 0.00001. The size – number of nodes respectively splits – of a tree controls whether interactions are fitted. With more complex trees fewer trees being required for minimum error, but the contribution of these interaction effects can be difficult to detect. Furthermore, the mintn affect the complexity of a tree, conditional on the number of observations in the trees terminal nodes [100]. For small samples there is no advantage for using large trees (high tc) [99]. Due to the small sample size for each ES in our analysis, we used a tc of 1 (decision “stump” of two terminal nodes) that fit simple additive effects and tested a mintn of 3, 5 and 8 respectively [100]. These tuning parameter then affecting the nt required for a reliable value transfer. For the identification of an optimal nt we used a three-fold

cross-validation, in accordance to [101]. With the determination of an optimal nt over-fitting models to training data can be reduced and their generality enhanced, consequently, the model performance improved when values are transferred to unsampled areas. The final parameters selected for the BRT models are shown in Fig S.3. Based on the optimized model parametrization for 12 ES types (839 monetary values) out of 22 (1033 monetary values) statistical significant value transfer functions could be computed (Fig 2) and most important covariates quantified (Figs 3 and S2). Data was not sufficient for generating value transfer functions if there were less than 11 studies or less than 26 data points. All covariates with more than 1% relative influence on BRT models were considered.

For the spatial extrapolation of values in unsampled areas we used the value transfer functions and applied them based on covariates of cells on a 30 arc min grid across the globe. Value transfer in geographic space was made by using scripts from “raster” library of R. Thus, we mapped for each ES type monetary values for the entire terrestrial earth surface (Figs 4 and S1).

To estimate the explanatory power of a value transfer function we computed the coefficient of determination R^2 for each value transfer function based on ten-fold cross-validation. Results are shown in Fig 3 (column 4).

Additionally, we examined each value transfer function for failure to generalize from training data in order to estimate the confidence intervals around transferred values. Therefore, the bias that coincided with poor nt is shown by assuming nt-vectors from 100 to 100.000. For each ES separate BRT models were fitted and the 2.5- and 97.5-percentile values of the variance of monetary value for each grid cell calculated as an estimate of the confidence intervals. Value transfer in geographic space was made in the same way as we mentioned above for spatial extrapolation of values in unsampled areas. Thereafter, we mapped the range of percentiles for each grid cell and grouped it into three classes (low, middle, high).The grouping was conducted by equal-interval classification for each ES separately, i.e. division of percentile ranges into classes that contain an equal range of values. Accordingly, ES crossing comparison are limited. The final bivariate maps (Figs 4 and S1) were developed by mapping the overlay of low, middle and high uncertainty with extrapolated monetary values from the optimized models. Also, based on these classes the percentage area of terrestrial earth surface covered by low, middle and high uncertainty was calculated; see Fig 3 (column 4).

Additional references

97. Eiswerth ME, Shaw WD. Adjusting benefits transfer values for inflation. Water Resources Research. 1997;33(10):2381-5. doi: 10.1029/97WR01983.
98. Friedman JH, Meulman JJ. Multiple additive regression trees with application in epidemiology. Stat Med. 2003;22(9):1365-81. doi: 10.1002/sim.1501. PubMed PMID: WOS:000182655100003.
99. Elith J, Leathwick JR, Hastie T. A working guide to boosted regression trees. J Anim Ecol. 2008;77(4):802-13. doi: 10.1111/j.1365-2656.2008.01390.x. PubMed PMID: WOS:000256539800020; PubMed Central PMCID: PMCBRT.
100. Hastie T, Tibshirani R, Friedman JH. The Elements of Statistical Learning: Data Mining, Inference, and Prediction: Springer; 2001.
101. Ridgeway G. Generalized boosted models: A guide to the gbm package2009:[12 p.]. Available from: http://bioconductor.wustl.edu/extra/vignettes/gbm/inst/doc/gbm.pdf.
