# Supplementary material for: Uncertainty of Monetary Valued Ecosystem Services – Value Transfer Functions for Global Mapping
Source: PLoS One. 2016 Mar 3;11(3):e0148524. doi: 10.1371/journal.pone.0148524 (PMC4777407; doi:10.1371/journal.pone.0148524)
Supplement: S2 Text — (PDF) [file pone.0148524.s007.pdf]

S1 References in Figures. The reference list links citations used in Figure 5.

1. Jackson LE, Pascual U, Hodgkin T. Utilizing and conserving agrobiodiversity in agricultural landscapes. Agriculture Ecosystems & Environment. 2007;121(3):196-210. doi: 10.1016/j.agee.2006.12.017. PubMed PMID: WOS:000245685900002.
2. Akter S, Bennett J, Akhter S. Preference uncertainty in contingent valuation. Ecological Economics. 2008;67(3):345-51. doi: 10.1016/j.ecolecon.2008.07.009. PubMed PMID: WOS:000260243000001.
3. Kontoleōn A, Macrory R, Swanson TM. Individual preference-based values and environmental decision making : should valuation have its day in court? Research in law and economics. 2002;20:177-214.
4. de Groot R, Brander L, van der Ploeg S, Costanza R, Bernard F, Braat L, et al. Global estimates of the value of ecosystems and their services in monetary units. Ecosystem Services. 2012;1(1):50-61. doi: http://dx.doi.org/10.1016/j.ecoser.2012.07.005. PubMed Central PMCID: PMCvaluation.
5. Seppelt R, Dormann CF, Eppink FV, Lautenbach S, Schmidt S. A quantitative review of ecosystem service studies: approaches, shortcomings and the road ahead. J Appl Ecol. 2011;48(3):630-6. doi: 10.1111/j.1365-2664.2010.01952.x. PubMed PMID: WOS:000290587100015; PubMed Central PMCID: PMCdatabase.
6. Rosenberger RS, Stanley TD. Measurement, generalization, and publication: Sources of error in benefit transfers and their management. Ecological Economics. 2006;60(2):372-8. doi: 10.1016/j.ecolecon.2006.03.018. PubMed PMID: WOS:000242976100005; PubMed Central PMCID: PMCbenefit transfer, accuracy.
7. Nelson J, Kennedy P. The Use (and Abuse) of Meta-Analysis in Environmental and Natural Resource Economics: An Assessment. Environ Resour Econ. 2009;42(3):345-77. doi: 10.1007/s10640-008-9253-5. PubMed PMID: WOS:000263504600004; PubMed Central PMCID: PMCmeta-analysis.
8. Bennett ND, Croke BFW, Guariso G, Guillaume JHA, Hamilton SH, Jakeman AJ, et al. Characterising performance of environmental models. Environ Modell Softw. 2013;40:1-20. doi: 10.1016/j.envsoft.2012.09.011. PubMed PMID: WOS:000314074000001.
9. Kirchner JW, Hooper RP, Kendall C, Neal C, Leavesley G. Testing and validating environmental models. Sci Total Environ. 1996;183(1-2):33-47. doi: 10.1016/0048-9697(95)04971-1. PubMed PMID: WOS:A1996UH96300005.
10. Spiegelhalter DJ, Riesch H. Don't know, can't know: embracing deeper uncertainties when analysing risks. Philos Trans R Soc A-Math Phys Eng Sci. 2011;369(1956):4730-50. doi: 10.1098/rsta.2011.0163. PubMed PMID: WOS:000296591000005; PubMed Central PMCID: PMCUncertainty.
11. Keynes JM. The general theory of employment. The Quarterly Journal of Economics. 1937;51(2):209-23. doi: doi: 10.2307/1882087.
12. Costanza R, de Groot R, Sutton P, van der Ploeg S, Anderson SJ, Kubiszewski I, et al. Changes in the global value of ecosystem services. Global Environmental Change. 2014;26(0):152-8. doi: http://dx.doi.org/10.1016/j.gloenvcha.2014.04.002. PubMed Central PMCID: PMCvaluation.
13. Costanza R, dArge R, deGroot R, Farber S, Grasso M, Hannon B, et al. The value of the world's ecosystem services and natural capital. Nature. 1997;387(6630):253-60. doi: 10.1038/387253a0. PubMed PMID: WOS:A1997WZ16700043.
14. Eigenbrod F, Armsworth PR, Anderson BJ, Heinemeyer A, Gillings S, Roy DB, et al. Error propagation associated with benefits transfer-based mapping of ecosystem services. Biol Conserv. 2010;143(11):2487-93. doi: http://dx.doi.org/10.1016/j.biocon.2010.06.015. PubMed Central PMCID: PMCbenefit transfer uncertainty.
15. Konarska KM, Sutton PC, Castellon M. Evaluating scale dependence of ecosystem service valuation: a comparison of NOAA-AVHRR and Landsat TM datasets. Ecological Economics.

2002;41(3):491-507. doi: 10.1016/s0921-8009(02)00096-4. PubMed PMID: WOS:000177073400010.
16. Bateman IJ, Day BH, Georgiou S, Lake I. The aggregation of environmental benefit values: Welfare measures, distance decay and total WTP. Ecological Economics. 2006;60(2):450-60. doi: 10.1016/j.ecolecon.2006.04.003. PubMed PMID: WOS:000242976100013.
17. Saenz-Arroyo A, Roberts CM, Torre J, Carino-Olvera M, Enriquez-Andrade RR. Rapidly shifting environmental baselines among fishers of the Gulf of California. Proc R Soc B-Biol Sci. 2005;272(1575):1957-62. doi: 10.1098/rspb.2005.3175. PubMed PMID: WOS:000231799900013.
